# Sequential Crystallization and Multicrystalline Morphology in PE-*b*-PEO-*b*-PCL-*b*-PLLA Tetrablock Quarterpolymers

**DOI:** 10.1021/acs.macromol.1c01186

**Published:** 2021-07-23

**Authors:** Eider Matxinandiarena, Agurtzane Múgica, Agnieszka Tercjak, Viko Ladelta, George Zapsas, Nikos Hadjichristidis, Dario Cavallo, Araceli Flores, Alejandro J. Müller

**Affiliations:** † POLYMAT and Department of Polymers and Advanced Materials: Physics, Chemistry and Technology, University of the Basque Country UPV/EHU, Paseo Manuel Lardizábal 3, 20018 Donostia-San Sebastián, Spain; ‡ Group ‘Materials + Technologies’, Department of Chemical and Environmental Engineering, University of the Basque Country, UPV/EHU, Plaza Europa 1, 20018 Donostia-San Sebastián, Spain; § Polymer Synthesis Laboratory, KAUST Catalysis Center, Physical Sciences and Engineering Division, King Abdullah University of Science and Technology (KAUST), Thuwal 23955-6900, Saudi Arabia; ∥ Department of Chemistry and Industrial Chemistry, University of Genova, via Dodecaneso 31, 16146 Genova, Italy; ⊥ Polymer Physics, Elastomers and Applications Energy, Institute of Polymer Science and Technology (ICTP-CSIC), Juan de la Cierva 3, 28006 Madrid, Spain; # Ikerbasque, Basque Foundation for Science, Plaza Euskadi 5, 48009 Bilbao, Spain

## Abstract

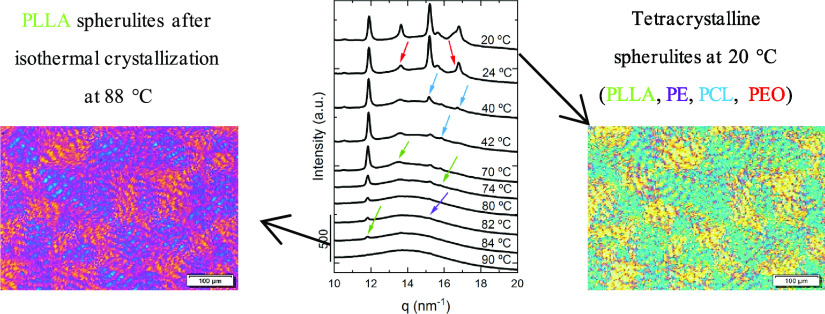

We investigate for the first time the morphology and crystallization of two novel tetrablock quarterpolymers of polyethylene (PE), poly(ethylene oxide) (PEO), poly(ε-caprolactone) (PCL), and poly(l-lactide) (PLLA) with four potentially crystallizable blocks: PE_18_^7.1^*-b-*PEO_37_^15.1^*-b-*PCL_26_^10.4^*-b-*PLLA_19_^7.6^ (Q1) and PE_29_^9.5^*-b-*PEO_26_^8.8^*-b-*PCL_23_^7.6^*-b-*PLLA_22_^7.3^ (Q2) (superscripts give number average molecular weights in kg/mol, and subscripts give the composition in wt %). Their synthesis was performed by a combination of polyhomologation (C1 polymerization) and ring-opening polymerization techniques using a ″catalyst-switch″ strategy, either ″organocatalyst/metal catalyst switch″ (Q1 sample, 96% isotactic tetrads) or ″organocatalyst/organocatalyst switch″ (Q2 sample, 84% isotactic tetrads). Their corresponding precursors—triblock terpolymers PE*-b-*PEO*-b-*PCL, diblock copolymers PE*-b-*PEO, and PE homopolymers—were also studied. Cooling and heating rates from the melt at 20 °C/min were employed for most experiments: differential scanning calorimetry (DSC), polarized light optical microscopy (PLOM), *in situ* small-angle X-ray scattering/wide-angle X-ray scattering (SAXS/WAXS), and atomic force microscopy (AFM). The direct comparison of the results obtained with these different techniques allows the precise identification of the crystallization sequence of the blocks upon cooling from the melt. SAXS indicated that Q1 is melt miscible, while Q2 is weakly segregated in the melt but breaks out during crystallization. According to WAXS and DSC results, the blocks follow a sequence as they crystallize: PLLA first, then PE, then PCL, and finally PEO in the case of the Q1 quarterpolymer; in Q2, the PLLA block is not able to crystallize due to its low isotacticity. Although the temperatures at which the PEO and PCL blocks and the PE and PLLA blocks crystallize overlap, the analysis of the intensity changes measured by WAXS and PLOM experiments allows identifying each of the crystallization processes. The quarterpolymer Q1 remarkably self-assembles during crystallization into tetracrystalline banded spherulites, where four types of different lamellae coexist. Nanostructural features arising upon sequential crystallization are found to have a relevant impact on the mechanical properties. Nanoindentation measurements show that storage modulus and hardness of the Q1 quarterpolymer significantly deviate from those of the stiff PE and PLLA blocks, approaching typical values of compliant PEO and PCL. Results are mainly attributed to the low crystallinity of the PE and PLLA blocks. Moreover, the Q2 copolymer exhibits inferior mechanical properties than Q1, and this can be related to the PE block within Q1 that has thinner crystal lamellae according to its much lower melting point.

## 1 Introduction

The crystallization of multiphasic block copolymers is a complex process that depends on several variables: segregation strength, composition, molecular weight, and thermal protocol applied during crystallization. Several reviews and many publications have been devoted to these materials due to their versatility and possible applications in several areas, including nanotechnology.^1−5^

Multicrystalline block polymers can consist of multiple crystallizable blocks. Several works have been published about AB-type diblock copolymers with one or two crystallizable blocks, such as PE*-b-*PLLA,^6−12^ PE*-b-*PEO,^2,13−18^ PE*-b-*PCL,^19−22^ PEO*-b-*PCL,^23−30^ PEO*-b-*PLLA,^31−37^ and PCL*-b-*PLA^38−43^ diblock copolymers.

The crystallization behavior becomes even more complex when a third block is considered. Some ABC-type tricrystalline terpolymers have been investigated due to their interesting properties.^5,44−49^ Most of the studies have been carried out employing biocompatible and/or biodegradable blocks, such as polyethyelene, poly(ethylene oxide) (PEO), poly(l-lactide) (PLLA), and poly(ε-caprolactone) (PCL), as they may have potential applications in biomedicine.^50,51^

Palacios et al.^48^ investigated PEO-*b-*PCL*-b-*PLLA triblock terpolymers, and after studying the different competitive effects such as nucleation, plasticization, antiplasticization, and confinement that took place within the blocks, they were able to show the triple crystalline nature of the samples by DSC and SAXS/WAXS experiments. Furthermore, triple crystalline spherulites were detected by PLOM; first, PLLA spherulitic templates were formed, and further cooling allowed the PCL and PEO blocks to crystallize within the interlamellar regions of the previously formed PLLA templates.

Sun et al.^50^ prepared PLLA*-b-*PCL*-b-*PEO*-b-*PCL*-b-*PLLA pentablock terpolymers. They demonstrated the coexistence of the three crystalline structures by DSC and WAXS experiments, although the crystallization of the central PCL block was hindered by the crystallization of the other PEO and PLLA blocks. On the other hand, Tamboli et al.^52^ only demonstrated crystallization of the PCL and PLLA blocks by WAXS in a PLLA*-b-*PCL*-b-*PEO*-b-*PCL*-b-*PLLA pentablock terpolymer.

Triblock terpolymers with an apolar polyethylene (PE) block have also been studied, for instance, by Vivas et al.^26^ They reported results for the triblock terpolymer polyethylene*-b-*poly(ethylene oxide)*-b-*poly(ε-caprolactone) (PE*-b-*PEO*-b-*PCL). Although they demonstrated the crystallization of the PE and PCL blocks, the PEO block was not able to crystallize in the synthesized material. The topological restrictions caused by the crystallization of the other two blocks were the main factors that prevented the crystallization of the PEO block.

Regarding ABC-type terpolymers with an apolar PE block, Müller et al.^53^ investigated the triple crystalline behavior of PE*-b-*PCL*-b-*PLLA and PE*-b-*PEO*-b-*PLLA triblock terpolymers. Although the crystallization of the PE and PLLA block occurs in a similar temperature range, they were able to show by WAXS the crystallization of all blocks in both materials, proving the triple crystalline nature of the samples. In addition, they studied the effect of the cooling rate since they discovered that the block crystallization sequence changed in the PE*-b-*PCL*-b-*PLLA triblock terpolymer. The first block to crystallize was the PE block using 20 °C/min as the cooling rate, whereas when the cooling rate was changed to 1 °C/min, the PLLA block was the first one to crystallize. This change in the crystallization sequence has an effect on the morphology, and thus, properties could be tuned by controlling cooling conditions to design novel materials.

The synthesis of well-defined tetracrystalline tetrablock quarterpolymers is a challenge, and to the best of our knowledge, there is only one report about this ABCD-type material. Hadjichristidis et al.^54^ reported a one-pot synthesis of tetracrystalline tetrablock quarterpolymer poly(ethylene)-*b*-poly(ethylene oxide)-*b*-poly(ε-caprolactone)-*b*-poly(l-lactide) (PE-*b*-PEO-*b*-PCL-*b*-PLLA) from a PE-OH macroinitiator by an organic/organic or organic/metal ″catalyst switch″ strategy. The formation of a tetrablock quarterpolymer was confirmed by ^1^H NMR spectroscopy (in liquid and solid state) and gel-permeation chromatography.

In this work, the crystallization behavior of the novel tetracrystalline tetrablock quarterpolymer PE-*b*-PEO-*b*-PCL-*b*-PLLA is studied. Two different block compositions are considered, varying the block content and molecular weight of each of the blocks (quarterpolymer Q1 and Q2). Their synthesis was performed by a combination of polyhomologation (C1 polymerization) and ring-opening polymerization techniques using a ″catalyst-switch″ strategy, either ″organocatalyst/metal catalyst switch″ (first sample, 98% isotactic tetrads) or ″organocatalyst/organocatalyst switch″ (second samples, 84% isotactic tetrads). Their precursors (triblock terpolymers, diblock copolymers, and homopolymers) are also studied for comparison purposes. We study for the first time the ability of all the blocks to crystallize in these complex materials. The influence of the restrictions imposed during the crystallization on the morphology and the final lamellar structure will be explored and correlated with the mechanical properties measured by nanoindentation. This study is carried out employing differential scanning calorimetry (DSC), *in situ* small-angle and wide-angle X-ray scattering (SAXS/WAXS) measurements, polarized light optical microscopy (PLOM), atomic force microscopy (AFM), and nanoindentation. These characterization techniques allow performing a comprehensive investigation of the crystalline behavior of these novel materials and the impact on relevant properties such as mechanical ones. The understanding of the complex crystalline nature is vital to tune properties and design new interesting materials for potential applications.

## 2 Experimental Section

### 2.1 Materials

**Scheme 1:**
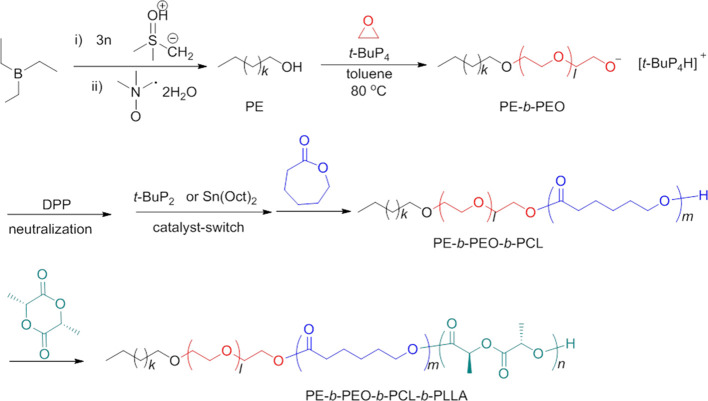
Synthesis of Tetracrystalline Quarterpolymer PE-*b*-PEO-*b*-PCL-*b*-PLLA by a Combination of Polyhomologation and the ″Catalyst Switch″ Strategy^54^

The poly(ethylene)*-b-*poly(ethylene oxide)*-b-*poly(ε-caprolactone)*-b-*poly(l-lactide) (PE*-b-*PEO*-b*-PCL*-b-*PLLA) quarterpolymers were obtained by one-pot synthesis using a ″catalyst switch″ strategy, either organic/organic or organic/metal (Table 1 and Scheme 1).

**Table 1:** Block Molecular Weight (*M_n_*) of the Homopolymers, Diblock Copolymers, Triblock Terpolymers, and Tetrablock Quarterpolymers

sample^e^	*M*_n_^c^ PE (g/mol)	*M*_n_^c^ PEO (g/mol)	*M*_n_^c^ PCL (g/mol)	*M*_n_^c^ PLLA (g/mol)	PDI^d^
PE^7.1^	7100				1.32
PE_32_^7.1^*-b-*PEO_68_^15.1^	7100	15,100			
PE_22_^7.1^*-b-*PEO_46_^15.1^*-b-*PCL_32_^10.4^	7100	15,100	10,400		
PE_18_^7.1^*-b-*PEO_37_^15.1^*-b-*PCL_26_^10.4^*-b-*PLLA_19_^7.6^ (Q1)^a^	7100	15,100	10,400	7600	
PE^9.5^	9500				1.28
PE_52_^9.5^*-b-*PEO_48_^8.8^	9500	8800			
PE_37_^9.5^*-b-*PEO_34_^8.8^*-b-*PCL_29_^7.6^	9500	8800	7600		
PE_29_^9.5^*-b-*PEO_26_^8.8^*-b-*PCL_23_^7.6^*-b-*PLLA_22_^7.3^ (Q2)^b^	9500	8800	7600	7300	

a Q1 was synthesized by the organic/metal ″catalyst switch″ (*t*-BuP_4_/DPP/Sn[Oct]_2_) strategy (isotactic tetrads 98%).

b Q2 was synthesized by the organic/organic ″catalyst switch″ (*t*-BuP_4_/DPP/*t*-BuP_2_) strategy (isotactic tetrads 84%).

c Determined by a 600 MHz ^1^H NMR spectrometer from the isolated polymer (toluene *d*_8_, 80 °C).

d Determined by high-temperature GPC in 1,2,4-trichlorobenzene at 150 °C (PS standards).

e Subscripts denote composition in wt %, and superscripts denote *M_n_* values in kg/mol.

A linear hydroxyl-terminated polyethylene (PE-OH) was firstly synthesized by polyhomologation of dimethylsulfoxonium methylide with triethyl borane as the initiator/catalyst^55^ and used with *t*-BuP_4_ (catalyst) to promote the ring-opening polymerization (ROP) of ethylene oxide (EO) toward PE-*b*-PEO. Then, neutralization of *t*-BuP_4_ was carried out with diphenyl phosphate (DPP), and a weaker base *t*-BuP_2_ was added to catalyze the ROP of ε-caprolactone (CL) and l-lactide (LLA) in toluene at 80 °C. The addition of this weaker *t-*BuP_2_ (organic/organic ″catalyst switch″) avoids as much as possible side reactions, although they are not completely suppressed. However, under these conditions, *S,R-*lactide monomeric units are formed because of racemization, which leads to a decrease of PLLA crystallinity. Therefore, an organic/metal *t-*BuP_4_/DPP/Sn(Oct)_2_ ″catalyst switch″ strategy was applied, which consists of using tin(II) 2-ethylhexanoate [Sn(Oct)_2_] to obtain *S,S-*lactide monomeric units.^54^

Table 1 reports the molecular weights of the synthesized materials. The subscript numbers give the composition in wt %, and the superscripts represent *M*_n_ values in kg/mol. This paper is mainly focused on the analysis of tetrablock quarterpolymers due to their novelty as tetracrystalline materials, although some results of the precursors are provided in the Supporting Information to have a complete overview of the crystalline behavior of these materials.

Due to monomer purity and possible side reactions, the polyethylene block precursors are not 100% linear. We perform NMR tests, and the results indicate that the PE block of Q1 contains 0.32% propyl side groups and 3% methyl groups while that of Q2 contains 0.45% propyl side groups and 2% methyl groups. This difference in microstructure explains their different melting points since the *T*_m_ value of PE^7.1^ is 130 °C (see Figure S1 and Table S3) while that of PE^9.5^ is 117 °C (see Figure S2 and Table S3), as this last material contains a higher amount of short-chain branches.

The formation of tetracrystalline quarterpolymers was confirmed by ^1^H NMR spectroscopy and gel-permeation chromatography.^54^ Furthermore, differential scanning calorimetry (DSC), polarized light optical microscopy (PLOM), and X-ray diffraction (SAXS/WAXS) proved the existence of different crystalline domains depending on the sample analyzed, as will be shown below.

### 2.2 Differential Scanning Calorimetry (DSC)

A Perkin Elmer DSC Pyris 1 calorimeter with an Intracooler 2P (cooling device) was employed to perform nonisothermal DSC experiments. Indium and tin standards were used for calibration. About 3 mg of sample was used after encapsulation in standard aluminum pans. An ultra-high-purity nitrogen atmosphere was employed.

Nonisothermal experiments were run in a temperature range between 0 and 180 °C or 0 and 160 °C, depending on the samples under study to avoid degradation, at 20 °C/min as the cooling and heating rate. Thermal history is erased by keeping the samples for 3 min at 30 °C above the peak melting temperature of the highest temperature melting block; samples are then cooled down, keeping them for 1 min at low temperatures to stabilize the system, and then heated up at 20 °C/min.

### 2.3 Small-Angle and Wide-Angle X-ray Scattering (SAXS/WAXS)

Small-angle X-ray scattering (SAXS) and wide-angle X-ray scattering (WAXS) experiments were measured simultaneously at beamline BL11-NCD in the ALBA Synchrotron (Barcelona, Spain). Capillaries were employed to place samples in the beam path. A THMS600 Linkam hot stage together with a liquid nitrogen cooling device was employed for temperature control and to heat and cool the samples. SAXS/WAXS diffractograms were recorded while copolymers crystallized and melted, using the same cooling and heating conditions employed in nonisothermal DSC experiments and thus having comparable results.

The X-ray energy source amounted to 12.4 keV (λ = 1.03 Å). For SAXS, a sample-detector distance of 6463 mm was used with a 0° tilt angle, and silver behenate was used for calibration (ADSC Q315r, Poway, CA, USA, with a resolution of 3070 × 3070 pixels and pixel size of 102 μm^2^). For WAXS, the sample-detector distance was 132.6 mm with a 21.2° tilt angle, and chromium(III) oxide was employed to do the calibration (Rayonix LX255-HS detector, Evanston, IL, USA, with a resolution of 1920 × 5760 pixels and pixel size of 44 μm^2^). Data were obtained as intensity versus scattering vector *q* = 4πsinθλ^–1^. The value of λ was 1.03 Å.

### 2.4 Polarized Light Optical Microscopy (PLOM)

The morphological study was performed with an Olympus BX51 polarized light optical microscope (PLOM). A THMS600 Linkam hot stage with a liquid N_2_ cooling device was used for temperature control. Images as well as videos were recorded with an SC50 (Olympus) camera. Samples were melted on a glass slide with a thin glass coverslip on top, and 20 °C/min was used as the cooling and heating rate to record all morphological changes. Furthermore, an isothermal experiment was also performed keeping the PE_18_^7.1^*-b-*PEO_37_^15.1^*-b-*PCL_26_^10.4^*-b-*PLLA_19_^7.6^ (Q1) quarterpolymer at 88 °C until the whole microscope field was covered with spherulites before applying a cooling scan at 20 °C/min.

In addition, the obtained micrographs were analyzed with ImageJ, an image processing software.^56^ The light intensity that passes through the cross polarizers in a sample is recorded, and an increase in that intensity means that the crystal content in the sample is increasing. The whole micrographs at different temperatures are considered as a ″region of interest″ to record intensity changes caused by all the superstructures that can be formed in the whole microscope field. This allows us to determine the temperature at which crystallization of a particular polymer block starts, and the whole crystallization process can be followed by means of intensity changes.

### 2.5 Atomic Force Microscopy (AFM)

The morphology of the samples was also explored by AFM. The observations were performed with a Bruker ICON scanning probe microscope equipped with a Nanoscope V controller. The micrographs were acquired in tapping mode using a TESP-V2 tip with a 127 μm cantilever (cantilever spring constant, *k* = 42 N/m, and resonance frequency, *f*_o_ = 320 kHz, Bruker). The AFM phase images of the investigated samples were subjected to a first-order plane-fitting procedure to compensate for the sample tilt.

Homogeneous thin film samples were spin-coated on mica substrates (SCC-200, Novocontrol Technologies, Germany) from tetrahydrofuran solutions (4 mg/mL) after determining the best sample preparation conditions. Then, different thermal protocols were applied on each sample before observing the samples at room temperature:

(a) Cooling from the melt at 50 °C/min to room temperature

(b) Cooling from the melt at 20 °C/min to room temperature

### 2.6 Nanoindentation

Samples were prepared on a Linkam hot plate by cooling (at 20 °C/min) from the melt to the crystallization temperature (*T*_c_) of each of the blocks (determined by DSC and WAXS as discussed in the manuscript) to perform isothermal steps of 5 min to crystallize each block until saturation before finally cooling down at 20 °C/min to room temperature. The coverslip was removed after the sample reached room temperature, and the glass slide was glued onto a cylindrical metal holder that was placed in the platform of a G200 nanoindenter (KLA Tencor, USA). A low load resolution head (dynamic contact module, DCM) with a Berkovich indenter was employed. The tip area was calibrated against a fused silica standard.^57^ During the loading ramp, a constant strain rate was employed (0.05 s^–1^), and at the same time, a small oscillating force at a frequency of 75 Hz was superimposed. Such dynamic testing allowed a continuous measure of the contact stiffness and damping during the loading cycle based on the phase lag between the oscillation force and the harmonic displacement.^58^ In the end, storage modulus, *E*′; loss modulus, *E*″; and hardness, *H*, were calculated.^57,58^ Poisson’s ratio was taken as 0.4. The advantage of dynamic testing with respect to quasi-static loading is twofold. On the one hand, time-dependent behavior can be examined. On the other, a profile of the mechanical properties as a continuous function of indentation depth can be obtained instead of a single reading at one indentation depth. As an example, Figure S3 illustrates the *E*′ and *H* behavior with indentation depth, *h*, for Q1 and Q2, and one can see that both mechanical properties are constant beyond *h ≈* 300 nm and up to the maximum penetration depth *h ≈* 1 μm. The fact that *E*′ and *H* remain constant with *h* suggests that mechanical properties are independent across the sample thickness and that substrate effects can be disregarded. Each *E*′ and *H* value of Figure S3 represents the average of at least 50 different indentations tests, and the error bars are associated to the standard deviation over the mean values. Indents were evenly distributed along the sample surface to make the average *E*′ and *H* values representative of the whole material. This is especially important in those samples in which the spherulite dimension was similar to the size of the volume of deformation (which approximately covers a hemisphere with a radius of 20 μm for an indentation depth of 1 μm^59^). For the case of PEO, the radii of the spherulites approached the millimeter scale and average *E*′ and *H* values were meaningless. Instead, a range of experimentally measured *E*′ and *H* values was reported.

## 3 Results and Discussion

### 3.1 Small-Angle X-ray Scattering (SAXS)

**Figure 1:**
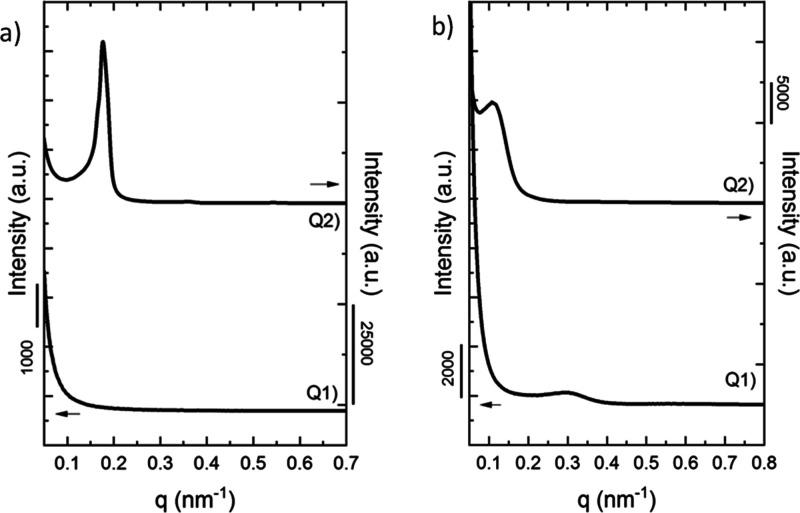
SAXS patterns of (Q1) PE_18_^7.1^*-b-*PEO_37_^15.1^*-b-*PCL_26_^10.4^*-b-*PLLA_19_^7.6^ and (Q2) PE_29_^9.5^*-b-*PEO_26_^8.8^*-b-*PCL_23_^7.6^*-b-*PLLA_22_^7.3^at (a) molten state at 180 °C indicating a lamellar structure in the melt by 1:2 *q* position of the scattering peaks and (b) room temperature of 25 °C.

SAXS measurements of all materials were employed to assess the possible phase segregation in the melt. Figure 1 shows the SAXS patterns, in which the intensity is plotted as a function of the scattering vector (*q*) of the tetrablock quarterpolymers in the molten state (Figure 1a) and at room temperature (Figure 1b). Since in the tetrablock PE_18_^7.1^*-b-*PEO_37_^15.1^*-b-*PCL_26_^10.4^*-b-*PLLA_19_^7.6^ (Q1) there are no diffraction peaks in the melt (Figure 1a, Q1), the only sample that is probably phase segregated in the melt is the tetrablock quarterpolymer PE_29_^9.5^*-b-*PEO_26_^8.8^*-b-*PCL_23_^7.6^*-b-*PLLA_22_^7.3^ (Q2) (Figure 1a, Q2) because of the presence of a sharp diffraction peak at low *q* values and a very weak second-order reflection located at 2*q* with respect to the first. Therefore, a lamellar phase segregation is most probably present in the Q2 melt. However, a crystallization breakout occurs (see the shift in *q* values between the sharp reflection in the melt and the weaker reflection at room temperature that appears at lower *q* values) when the sample is cooled down. The phase structure formed by phase segregation in the melt was probably destroyed and replaced by crystalline lamellae that scattered X-rays at lower *q* values. Breakout usually occurs when the phase segregation between block components is weak (Figure 1b, Q2). This weak phase segregation behavior was corroborated by the presence of small PE spherulites observed by PLOM in the Q2 quarterpolymer even when the PE content in the material is only 29%, as will be discussed below.

The segregation strength in diblock copolymers can be predicted by calculating the Flory–Huggins interaction parameter (χ) and multiplying it by *N*, the polymerization degree. However, the mean-field segregation theory was derived for diblock copolymers, and when analyzing triblock or tetrablock copolymers, the theoretical estimation of the segregation strength becomes more complicated. To our knowledge, the experimental determination of χ values for terpolymers or quarterpolymers has not been reported yet. Nevertheless, an approximate estimation for each pair of blocks has been calculated by using the solubility parameters of PE, PEO, PCL, and PLLA reported in the literature.^60,61^

If the segregation strength χ*N* is lower than or equal to 10, the diblock copolymers are miscible in the melt; if χ*N* is between 10 and 30, they are weakly segregated; if χ*N* is between 30 and 50, the segregation is intermediate; and if χ*N* > 50, the system is strongly segregated. The values do not fully represent the whole interactions in our samples, and as data in Table S1 in the Supporting Information show, there is a wide range in the obtained values. As previously mentioned, only one tetrablock studied here is phase segregated in the melt (Figure 1a, Q2), which suggests that the molecular weight of the blocks and composition affect the phase behavior due to the contribution of each pair of blocks to the segregation strength.

### 3.2 Nonisothermal Crystallization by DSC

**Figure 2:**
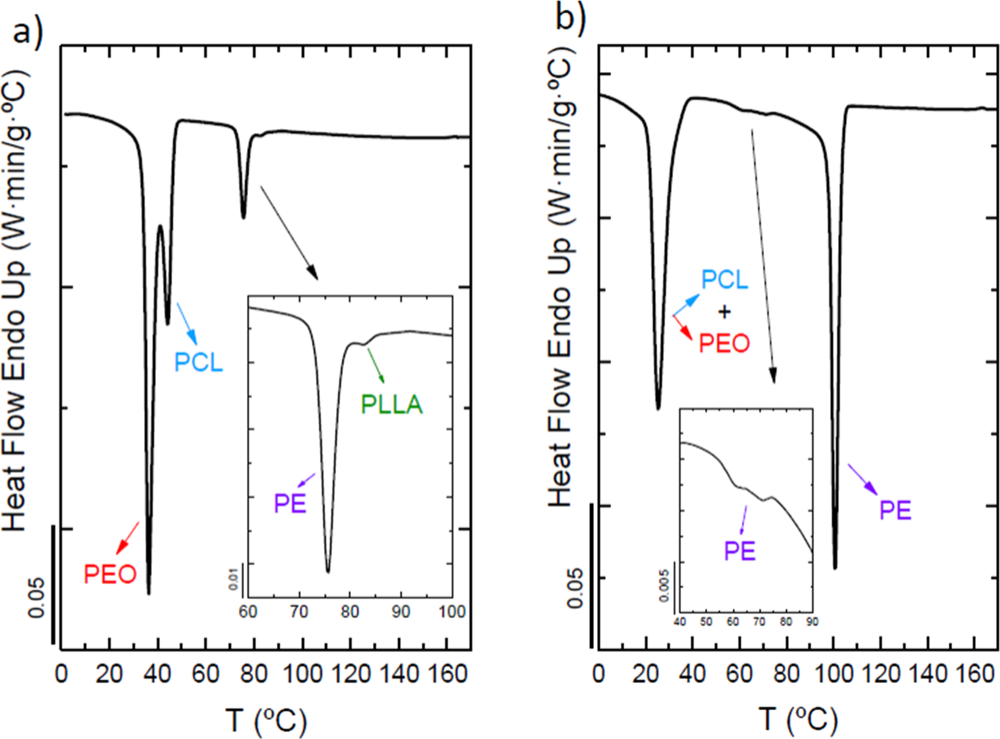
DSC cooling scans at 20 °C/min for tetrablock quarterpolymers (a) PE_18_^7.1^*-b-*PEO_37_^15.1^*-b-*PCL_26_^10.4^*-b-*PLLA_19_^7.6^ (Q1) and (b) PE_29_^9.5^*-b-*PEO_26_^8.8^*-b-*PCL_23_^7.6^*-b-*PLLA_22_^7.3^ (Q2) with arrows indicating transitions for each block (violet for PE, green for PLLA, blue for PCL, and red for PEO) and close-ups to better identify crystallization peaks.

Nonisothermal DSC scans were measured to analyze the crystallization of each block in the samples. DSC scans show that each block is able to crystallize, although some crystallization transitions overlap. DSC experiments upon cooling from the melt at 20 °C/min in Figure 2 show the exothermic crystallization peaks of the blocks of the corresponding tetrablock quarterpolymers: Figure 2a corresponds to PE_18_^7.1^*-b-*PEO_37_^15.1^*-b-*PCL_26_^10.4^*-b-*PLLA_19_^7.6^ (Q1), and Figure 2b corresponds to PE_29_^9.5^*-b-*PEO_26_^8.8^*-b-*PCL_23_^7.6^*-b-*PLLA_22_^7.3^ (Q2). The block content and molecular weight of each of the blocks (subscripts indicate composition in wt %, and superscripts indicate the number average molecular weight (*M*_n_) values in kg/ mol) are different in both tetrablock quarterpolymers (Q1 vs Q2). Crystallization (*T*_c_) of each of the blocks has been assigned by analyzing the WAXS data measured under identical cooling conditions (shown and described below).

Figure 2a shows the crystallization of all blocks, and the sequence is as follows: PLLA block, PE block, PCL block, and PEO block (colored arrows indicate crystallization peaks of each block: green for PLLA block, violet for PE block, blue for PCL block, and red for PEO block). Note that a close-up is inserted in Figure 2a to properly identify the crystallization exotherms of the PLLA and the PE blocks since both crystallizations are almost overlapped. However, this close-up clarifies that the PLLA block is the first block to crystallize at 84 °C followed by the PE block at 82 °C. This temperature value for the crystallization temperature of the PLLA block may seem to be too low, but WAXS measurements presented below confirm this crystallization sequence (Figures 4a and 5a). The crystallization of the PCL and PEO blocks occurs in the same temperature range; however, in this case, the very first peak at 42 °C corresponds to the PCL block followed by the crystallization of the PEO block at the lowest temperature, also confirmed by WAXS results in Figures 4a and 5a.

Figure 2b corresponds to the PE_29_^9.5^*-b-*PEO_26_^8.8^*-b-*PCL_23_^7.6^*-b-*PLLA_22_^7.3^ tetrablock quarterpolymer (Q2). In this case, the crystallization of the PLLA block does not occur, as the first block to crystallize is the PE block (violet arrow). In addition, a close-up shows the presence of another crystallization peak between 60 and 75 °C, which also corresponds to the crystallization of the PE block (also demonstrated by WAXS measurements in Figures 4b and 5b). This behavior is called fractionated crystallization, in which different crystallization events are observed for one component, the PE block in this case.^62^ Then, at lower temperatures, overlapped crystallizations of the PCL and the PEO block occur. We are not able to identify each of the crystallizations by DSC, but WAXS measurements below (Figures 4b and 5b) determine that the PCL block crystallizes a few degrees higher than the PEO block.

Figure 2a,b shows the effect of block content and molecular weight in the crystallization behavior since the same blocks constitute these two tetrablock quarterpolymers. Table 2 summarizes the crystallization ability of the blocks in these materials. In the first quarterpolymer (Q1), all blocks are able to crystallize. In the second quarterpolymer (Q2), the PLLA block does not crystallize due to its low isotacticity (see experimental part). Both the PLLA content (19–22) and the molecular weight are almost the same (7.3–7.6 kg/mol). So, the main difference in both quarterpolymers is the low isotacticity of the PLLA block within quarterpolymer Q2. In addition, the PE content in this Q2 quarterpolymer is higher than that in the other quarterpolymer Q1 (29 > 18), with almost the same molecular weight (9.5 > 7.1). The content of the other two blocks constituting the quarterpolymer does not vary significantly. So, these results show that the block nature mostly plays a key role in the crystallization behavior of complex quarterpolymers with four potentially crystallizable blocks, although block content may also affect the crystallization behavior.

**Table 2:**
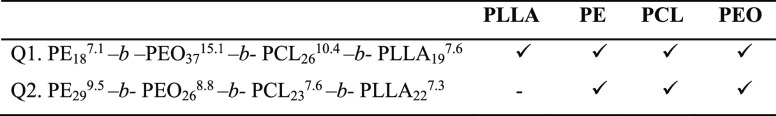
Tetrablock Quarterpolymers Q1 and Q2^a^

a The mark indicates the crystallization ability of each of the blocks. Subscripts indicate composition in wt %, and superscripts represent *M*_n_ values of each block in kg/mol.

**Figure 3:**
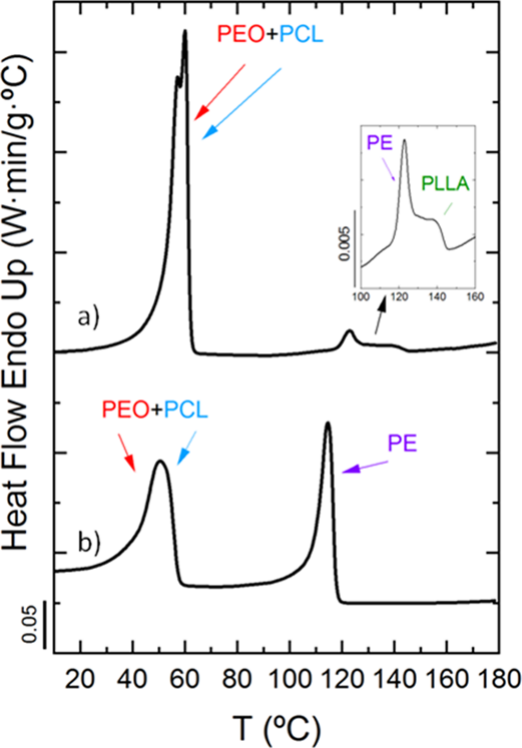
DSC heating scans at 20 °C/min for (a) PE_18_^7.1^*-b-*PEO_37_^15.1^*-b-*PCL_26_^10.4^*-b-*PLLA_19_^7.6^ (Q1) and (b) PE_29_^9.5^*-b-*PEO_26_^8.8^*-b-*PCL_23_^7.6^*-b-*PLLA_22_^7.3^ (Q2), with arrows indicating transitions for each block (violet for PE, green for PLLA, blue for PCL, and red for PEO) and a close-up to better identify melting peaks.

Figure 3 shows the subsequent DSC heating scans of the tetrablock quarterpolymers at 20 °C/min with the corresponding melting peaks (*T*_m_) of the blocks. Although, in both cases, the melting of the PEO and PCL blocks occurs in the same temperature range, the first block to melt is the PEO block followed by the PCL block according to WAXS studies. Then, the melting of the PE block occurs, and finally, the PLLA block is the last one to melt for Q1 (Figure 3, curve a) in which the PLLA and the PE block crystallize. A close-up in the range of 100–160 °C is inserted in the figure so that the melting transitions of both the PE and PLLA blocks can be clearly appreciated. In the other case, for the quarterpolymer Q2 (Figure 3b), the PLLA block does not melt as it cannot crystallize. All these transitions and the melting sequences are confirmed by WAXS measurements (Figure S4 in the Supporting Information).

In addition, all DSC data regarding the two quarterpolymers are collected in Tables S5–S7 in the Supporting Information since crystallization peak temperatures (*T*_c_) and enthalpies (Δ*H*_c_), melting peak temperatures (*T*_m_) and enthalpies (Δ*H*_m_), and crystallinity degrees of each of the blocks (*X*_c_) calculated from cooling and heating scans are provided. Note that cooling and heating transitions of the PE and PLLA blocks and PEO and PCL blocks overlap, and an estimation of the crystallinity values according to the block content is provided.

Furthermore, all the corresponding precursors of these Q1 and Q2 quarterpolymers listed in Table 1 have also been analyzed by DSC. DSC cooling and heating scans at 20 °C/min for the homopolymer PE^7.1^, the diblock copolymer PE_32_^7.1^*-b-*PEO_68_^15.1^, and the triblock terpolymer PE_22_^7.1^*-b-*PEO_46_^15.1^*-b-*PCL_32_^10.4^ are presented in Figure S1 (and relevant calorimetric data are reported in Tables S2–S4) in the Supporting Information, whereas the scans for the homopolymer PE^9.5^, the diblock copolymer PE_52_^9.5^*-b-*PEO_48_^8.8^, and the triblock terpolymer PE_37_^9.5^*-b-*PEO_34_^8.8^*-b-*PCL_29_^7.6^ are shown in Figure S2 of the Supporting Information (and relevant calorimetric data are reported in Tables S2–S4).

### 3.3 *In Situ* Wide-Angle X-ray Scattering (WAXS) Real-Time Synchrotron Results

WAXS data were compared to DSC results; both sets of experiments were performed employing the same cooling/heating rates. This is very advantageous since the direct comparison of DSC and WAXS results allows a better understanding of the crystallization sequence in these complex and novel tetrablock quarterpolymers.

The presence of crystalline reflections is pointed out in colors in all WAXS diffractograms presented below (violet for PE, green for PLLA, red for PEO, and blue for PCL), and it is confirmed that each block is able to crystallize separately. According to the literature, PLLA, PCL, and PE crystallize in orthorhombic unit^26,27,63^ cells and PEO in a monoclinic^27^ one. The crystal unit cell dimensions are the following: a = 10.56 Å, b = 6.05 Å, and c = 28.90 Å for PLLA;^63^ a = 7.48 Å, b = 4.98 Å, and c = 17.26 Å for PCL;^64^ a = 7.96 Å, b = 13.11 Å, c = 19.39 Å (chain direction), and β = 124°48′ for PEO;^65^ and a = 7.40 Å, b = 4.96 Å, and c = 2.53 Å for PE.^66^ All reflections observed in our samples correspond only to the α-form of PLLA; no signals were detected for the α′-form.^45^Table S8 in the Supporting Information reports the indexing that agrees well with assignments widely published in the literature for PE, PEO, PCL, and PLLA crystals.^27,39−41,45,60,63,67,68^

**Figure 4:**
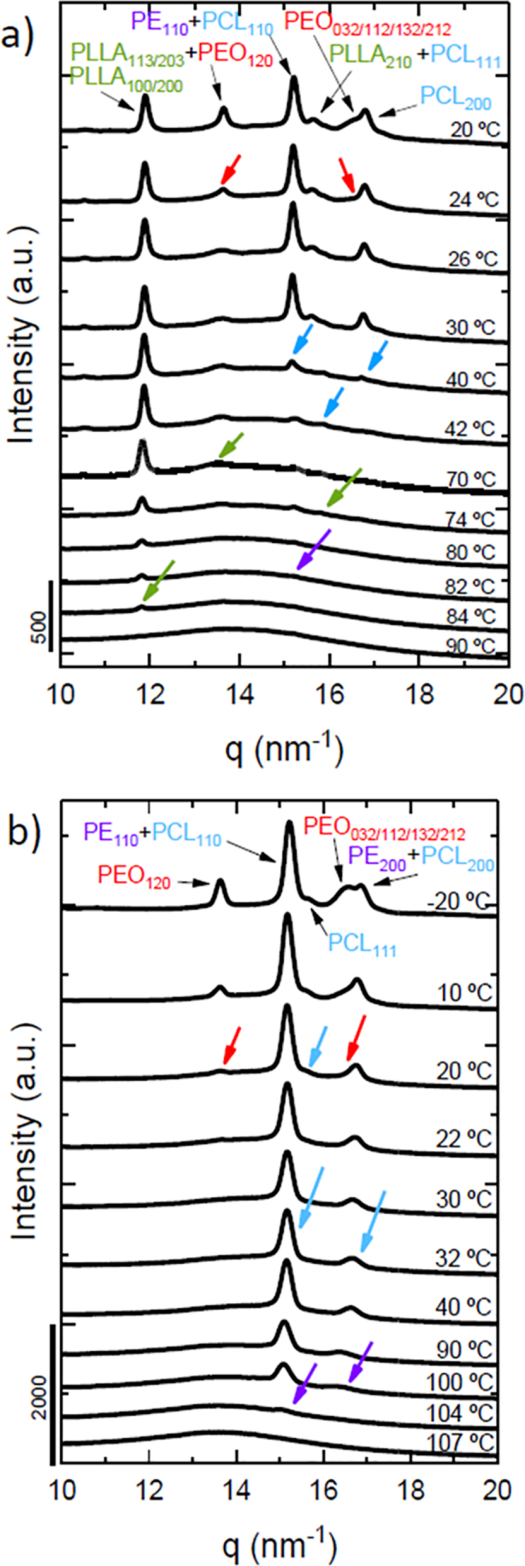
WAXS patterns taken during cooling from the melt at 20 °C/min for (a) PE_18_^7.1^*-b-*PEO_37_^15.1^*-b-*PCL_26_^10.4^*-b-*PLLA_19_^7.6^ (Q1) and (b) PE_29_^9.5^*-b-*PEO_26_^8.8^*-b-*PCL_23_^7.6^*-b-*PLLA_22_^7.3^ (Q2) at different temperatures with arrows indicating transitions for each block (violet for PE, green for PLLA, blue for PCL, and red for PEO) and the corresponding (hkl) planes of the blocks.

Figure 4 presents WAXS patterns upon cooling from the melt at 20 °C/min for the two tetrablock quarterpolymers analyzed in this work. In the tetrablock quarterpolymer represented in Figure 4a (Q1), all blocks are able to crystallize, and the crystallization sequence is the following: the PLLA block at 84 °C (green), the PE block at 82 °C (violet), the PCL block at 42 °C (blue), and finally the PEO block at 24 °C (red). We are able to determine this crystallization sequence due to the characteristic reflection peaks of each of the components: PLLA_110/200_ (*q* = 12.0 nm^–1^), PLLA_113/203_ (*q* = 13.5 nm^–1^), PEO_120_ (*q* = 13.8 nm^–1^), PE_110_ (*q* = 15.4 nm^–1^), PCL_110_ (*q* = 15.0 nm^–1^), PCL_111_ (*q* = 15.6 nm^–1^), PLLA_210_ (*q* = 15.7 nm^–1^), PEO_032/112/132/212_ (*q* = 16.4 nm^–1^), PCL_200_ (*q* = 16.7 nm^–1^), and PE_200_ (*q* = 16.9 nm^–1^). The presence of these scattering peaks at their corresponding *q* values corroborates the crystallization of each of the blocks.

In the same way, in Figure 4b, the scattering peaks of the corresponding blocks of the tetrablock quarterpolymer (Q2) are assigned with colored arrows. The first block to crystallize is the PE block at 104 °C (violet), followed by the PCL block (blue) at 32 °C, and finally by the PEO block (red) at 20 °C. These results confirm that the crystallization of the PLLA block in this sample does not occur, as its characteristic scattering peaks are not present in the WAXS patterns. These results confirm what was previously shown in the DSC scans in Figure 2b.

**Figure 5:**
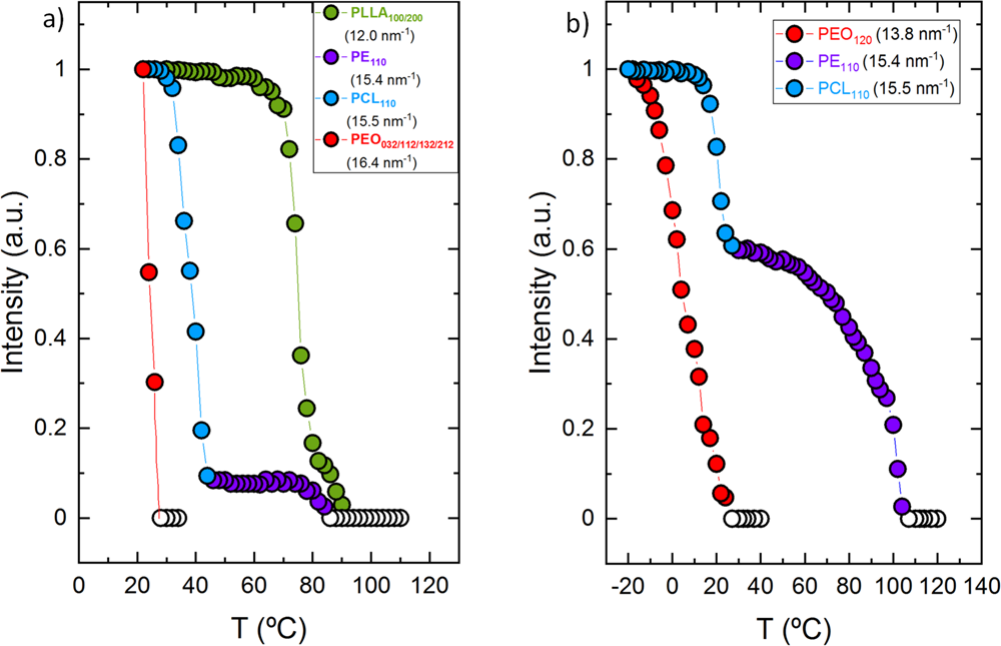
Normalized WAXS intensities as a function of the temperature of the indicated block reflections for (a) PE_18_^7.1^*-b-*PEO_37_^15.1^*-b-*PCL_26_^10.4^*-b-*PLLA_19_^7.6^ (Q1) and (b) PE_29_^9.5^*-b-*PEO_26_^8.8^*-b-*PCL_23_^7.6^*-b-*PLLA_22_^7.3^ (Q2) with colored data points and lines (violet for PE, green for PLLA, blue for PCL, and red for PEO) to follow the crystallization of each block. Empty data points represent the molten state of the corresponding block in the samples.

To determine the exact temperatures at which the crystallization of each of the blocks starts and the whole temperature range in which they crystallize, the normalized intensities of the scattering peaks as a function of temperature are plotted in Figure 5. The same color code employed previously is used to facilitate comprehension of the plots. Figure 5a shows the results for the PE_18_^7.1^*-b-*PEO_37_^15.1^*-b-*PCL_26_^10.4^*-b-*PLLA_19_^7.6^ tetrablock quarterpolymer (Q1), in which all four blocks are able to crystallize. The exclusive PLLA_100/200_ (green) and PEO_032_ (red) signals are employed to determine their crystallization ranges because these signals correspond only to PLLA or PEO crystals. The first sharp increase in the PLLA_100/200_ (green) signal corresponds to the PLLA block crystallization starting at 90 °C, whereas the increase starting at 24 °C in the PEO_032_ (red) confirms its crystallization. However, for the PE and PCL blocks, the joint reflection of PE_110_ (violet) and PCL_110_ (blue) is used, as there are no signals that correspond only to the PE or the PCL block. The first increase corresponds to the PE block crystallization starting at 82 °C, and the second sharp increase corresponds to the PCL block crystallization at 42 °C. The same methodology is employed for the PE_29_^9.5^*-b-*PEO_26_^8.8^*-b-*PCL_23_^7.6^*-b-*PLLA_22_^7.3^ (Figure 5b) tetrablock quarterpolymer (Q2) to determine the crystallization ranges of the blocks.

The WAXS subsequent heating transitions are reported in Figure S4 in the Supporting Information, which also confirm the presence of the crystalline blocks identified during cooling from the melt by WAXS (Figure 4).

### 3.4 Polarized Light Optical Microscopy (PLOM) Observations

Polarized light optical microscopy (PLOM) experiments allow studying the sequential crystallization of the blocks of the tetrablock quarterpolymers, as well as their superstructural organization. PLOM experiments have been performed using the same cooling and heating conditions employed in DSC and *in situ* WAXS experiments; thus, results are directly comparable, and the crystalline behavior and morphology of the materials can be determined.

**Figure 6:**
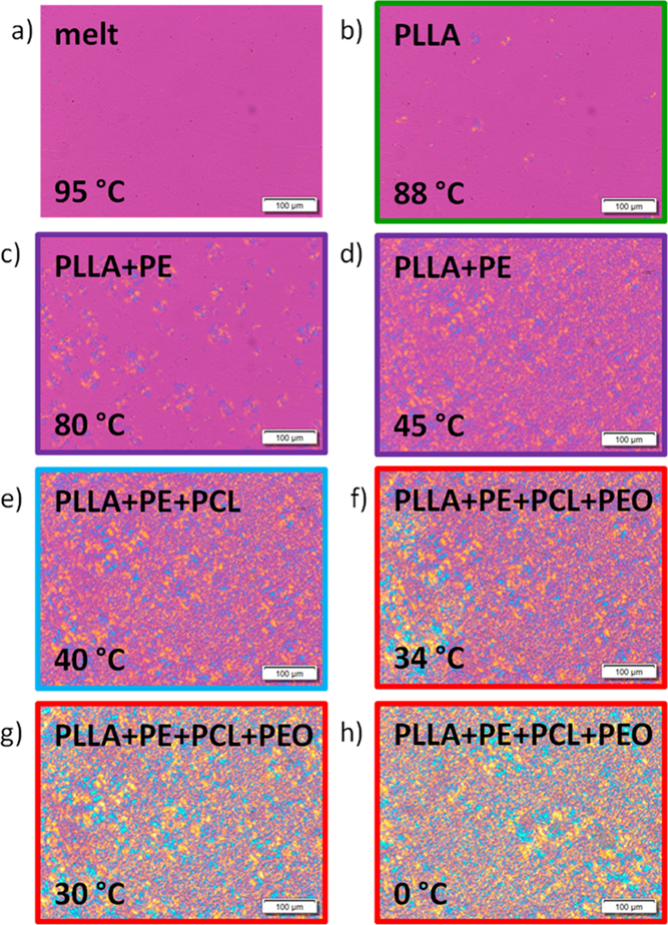
PLOM micrographs taken upon cooling from the melt at 20 °C/min with colored boxes indicating the crystallization of each of the blocks (violet for PE, green for PLLA, blue for PCL, and red for PEO) for the tetrablock quarterpolymer PE_18_^7.1^*-b-*PEO_37_^15.1^*-b-*PCL_26_^10.4^*-b-*PLLA_19_^7.6^ (Q1): (a) molten state at 95 °C; (b) PLLA at 88 °C; (c) PLLA and PE at 80 °C; (d) PLLA and PE at 45 °C; (e) PLLA, PE, and PCL at 40 °C; (f) PLLA, PE, PCL, and PEO at 34 °C; (g) PLLA, PE, PCL, and PEO at 30 °C; and (h) PLLA, PE, PCL, and PEO at 0 °C.

Figure 6 shows PLOM micrographs upon cooling from the melt at 20 °C/min for the PE_18_^7.1^*-b-*PEO_37_^15.1^*-b-*PCL_26_^10.4^*-b-*PLLA_19_^7.6^ tetrablock quarterpolymer (Q1). When the sample is in the molten state, at 95 °C (see micrograph a), there are no observable features. A legend on the top of the micrographs indicates the crystalline phases that should be present at the indicated temperatures according to the previously mentioned DSC and WAXS evidence. In addition, the same color code is employed to follow the crystallization of the different blocks.

Micrograph b (Figure 6) shows the first PLLA block spherulites at 88 °C; the small birefringent spots indicate that the crystallization of PLLA block crystals has started. After cooling down the sample to 80 °C in micrograph c (Figure 6), both PLLA block spherulites (which can already contain some PE lamellae within them) and smaller PE block spherulites grow simultaneously, as the crystallization of the PLLA block and the PE block is overlapped in a temperature range of approximately 70–90 °C (Figure 5a). We have a collection of PLLA block nucleated spherulites and PE block nucleated spherulites that may start at the same time but that eventually will contain both PLLA and PE crystalline lamellae within them; hence, they are double crystalline spherulites.

After cooling down the sample to 45 °C, the number of PLLA and PE nuclei has increased, as shown in micrograph d (Figure 6); PLLA nucleated spherulites and PE nucleated spherulites have grown at the same temperature range. Then, as crystallization of the PCL starts at 42 °C according to WAXS measurements (Figure 5a), a triple crystalline material is presented at 40 °C in micrograph e (Figure 6), with a wide size range of triple crystalline spherulites covering the entire microscope view field. PCL lamellae nucleate inside the PLLA- and PE-based spherulites.

At 34 °C, the crystallization of the PEO blocks starts, and it is evident since there is a clear change in the birefringence as shown in the left side of micrograph f (Figure 6), in addition to the WAXS results reported in Figure 5a. Micrograph g (Figure 6) shows that almost all the PEO block has already crystallized at 30 °C, as there are no more observable differences in birefringence upon cooling the sample to 0 °C in micrograph h (Figure 6). So, these PLOM micrographs show that the crystallization of all blocks has occurred, and tetracrystalline spherulites are finally obtained. As far as we are aware, this is the first time that a polymeric sample with tetracrystalline spherulites has been reported.

**Figure 7:**
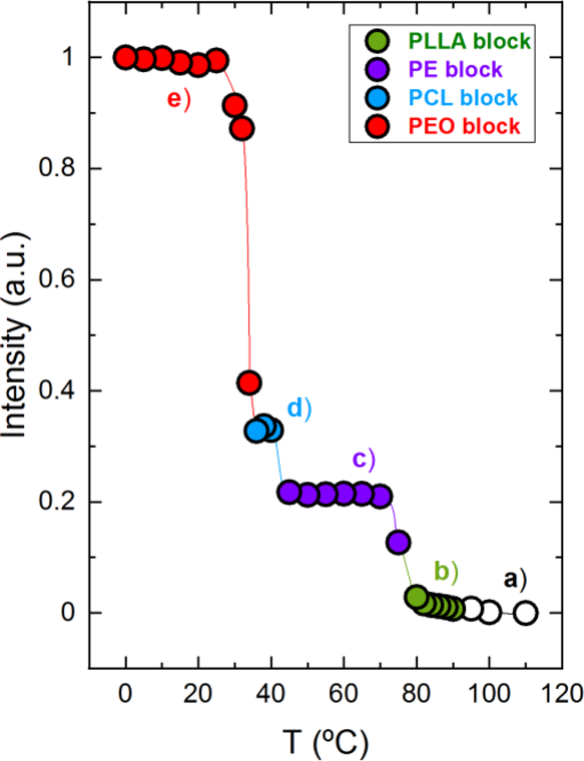
PLOM intensity measurements corresponding to the micrographs of Figure 6 as a function of temperature indicating (a) melt and the progressive crystallization upon cooling of the (b) PLLA block, (c) PE block, (d) PCL block, and (e) PEO block for the tetrablock quarterpolymer PE_18_^7.1^*-b-*PEO_37_^15.1^*-b-*PCL_26_^10.4^*-b-*PLLA_19_^7.6^ (Q1), with colored data points and lines (green for PLLA, violet for PE, blue for PCL, and red for PEO) to follow the crystallization of each block. Empty data points represent the molten state of the sample.

In addition, to properly analyze the crystallization of each of the blocks in the PLOM micrographs, light intensity measurements^56^ were performed. Figure 7 shows the recorded change in intensity as a function of temperature, with a–e colored letters of the micrographs in Figure 6 to see the corresponding morphology at those temperatures. Starting from the molten state (a), the first slight intensity increase corresponds to the crystallization of the PLLA block (b), followed by a sharper increase due to the PE block crystallization (c). Then, the crystallization of the PCL block increases the intensity value (d), and finally, the sharpest increase in intensity is due to the crystallization of the PEO block (e). This complements the micrographs shown in Figure 6 since it is hard to notice slight changes in intensity by human eyes, although the change caused by the crystallization of the PEO block is well noticeable in micrograph e in Figure 6.

**Figure 8:**
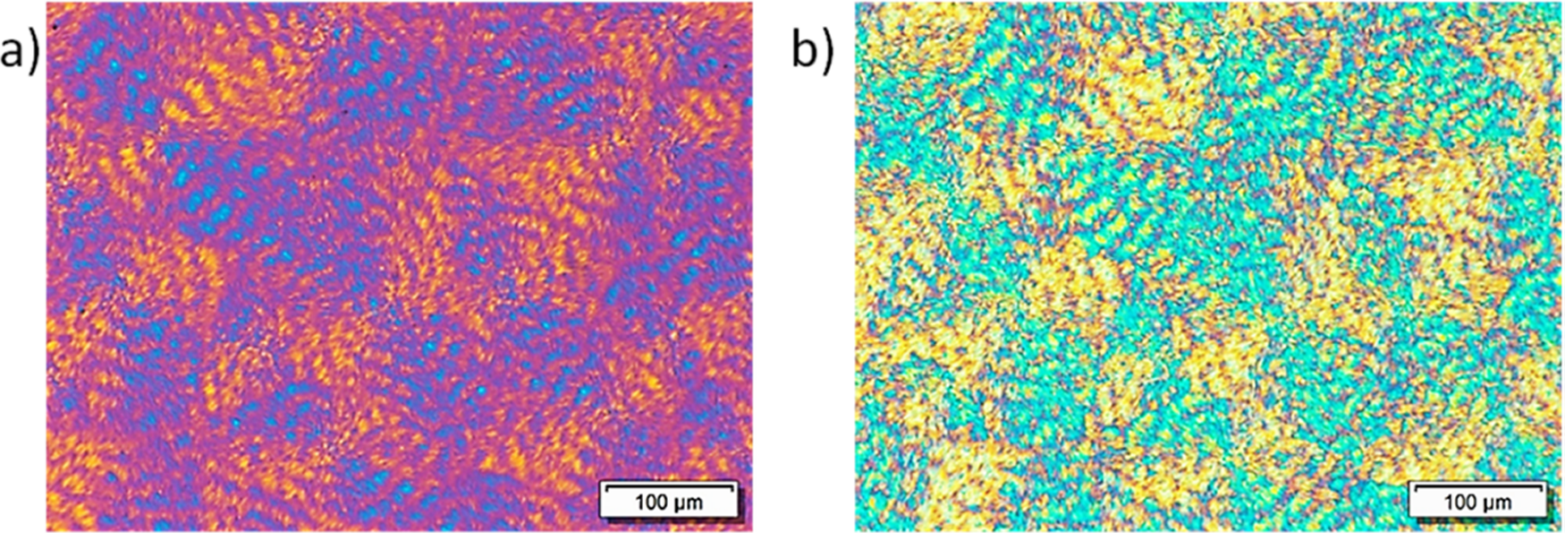
PLOM micrograph of the tetrablock quarterpolymer PE_18_^7.1^*-b-*PEO_37_^15.1^*-b-*PCL_26_^10.4^*-b-*PLLA_19_^7.6^ (Q1) (a) after isothermally crystallizing the sample at 88 °C during 40 min (a temperature at which only the PLLA block can crystallize) and (b) at room temperature after cooling the sample at 20 °C/min, allowing the crystallization of the other three blocks within the PLLA spherulites so that tetracrystalline spherulites are formed.

Furthermore, an additional measurement was performed with PLOM to see the morphology within larger spherulites than those obtained in Figure 6h. After melting the PE_18_^7.1^*-b-*PEO_37_^15.1^*-b-*PCL_26_^10.4^*-b-*PLLA_19_^7.6^ (Q1) quarterpolymer, an isothermal step at 88 °C was performed for 40 min to let the PLLA block crystallize until saturation, forming large spherulites (Figure 8a). The whole microscope field was filled with PLLA spherulites that are much larger than those obtained during the nonisothermal experiment discussed above (Figure 6h). These PLLA block spherulites can be considered a template partially filled with PLLA block crystalline lamellae (notice that the PLLA content is only 19% and not all of this material can crystallize), and the rest is composed by amorphous chains of all the tetrablock constituents (i.e., PLLA, PE, PCL, and PEO). It is remarkable that these spherulitic templates can display Maltese crosses and a negative sign, indicating that the PLLA chains are tangential to the spherulitic radius and also a banding extinction pattern.

Once complete crystallization of the PLLA block occurred at 88 °C, the sample was cooled down at 20 °C/min to room temperature, allowing the rest of the blocks of the quarterpolymer (the PE, PCL, and PEO blocks) to crystallize at their corresponding crystallization temperatures and finally obtaining the morphology shown in Figure 8b. The clear change in birefringence corroborates the crystallization of the last block (the PEO block, as discussed above in Figure 6), but since the PE and PCL blocks also crystallize during cooling, the final morphology corresponds to tetracrystalline spherulites that also display Maltese crosses, negative signs, and banding patterns. Such typical spherulitic characteristics probably indicate that the spherulite is composed of four types of lamellar crystals that grow radially within the PLLA template skeleton. The inner lamellar morphology of the spherulites was visualized by AFM (see below).

In addition, subsequent heating after quenching the quarterpolymer Q1 was performed to corroborate these results, and analogous observations were recorded. For more details, additional results are shown in Figures S5 and S6 in the Supporting Information.

**Figure 9:**
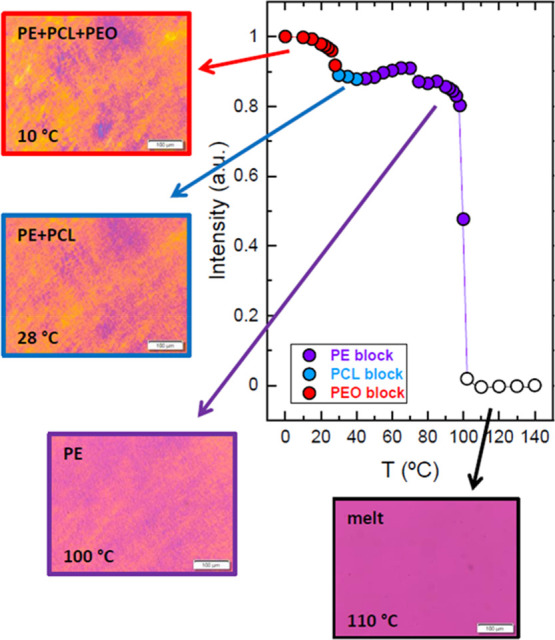
PLOM intensity measurements as a function of temperature with micrographs of cooling from the melt at 20 °C/min at the indicated temperatures with colored arrows and data points indicating the crystallization of each block for the tetrablock quarterpolymer PE_29_^9.5^*-b-*PEO_26_^8.8^*-b-*PCL_23_^7.6^*-b-*PLLA_22_^7.3^ (Q2) (violet for PE, blue for PCL, and red for PEO).

In Figure 9, light intensity measurements and PLOM micrographs of the tetrablock quarterpolymer PE_29_^9.5^*-b-*PEO_26_^8.8^*-b-*PCL_23_^7.6^*-b-*PLLA_22_^7.3^ (Q2) are presented. Note that the PLLA block does not crystallize according to DSC/WAXS results (Figures 2b and 4b), and the analysis of the light intensity corroborates that crystallization of the PLLA does not occur (Figure 9). The first sharp increase at approximately 100 °C corresponds to the crystallization of the PE block, and the pattern that shows the PLOM micrographs at 100 °C confirms so, as the PE block precursor has also been measured and found to have a very high nucleating density that leads to a microspherulitic morphology (not shown). Cooling down the sample crystallization of the PCL block happens at 32 °C, and the change in birefringence is evident in the micrograph shown at 28 °C. Then, the crystallization of the PEO block that starts at 20 °C is recorded by the increase in intensity as well as in the brightness of the micrograph at 10 °C (Figure 9).

### 3.5 Atomic Force Microscopy (AFM)

**Figure 10:**
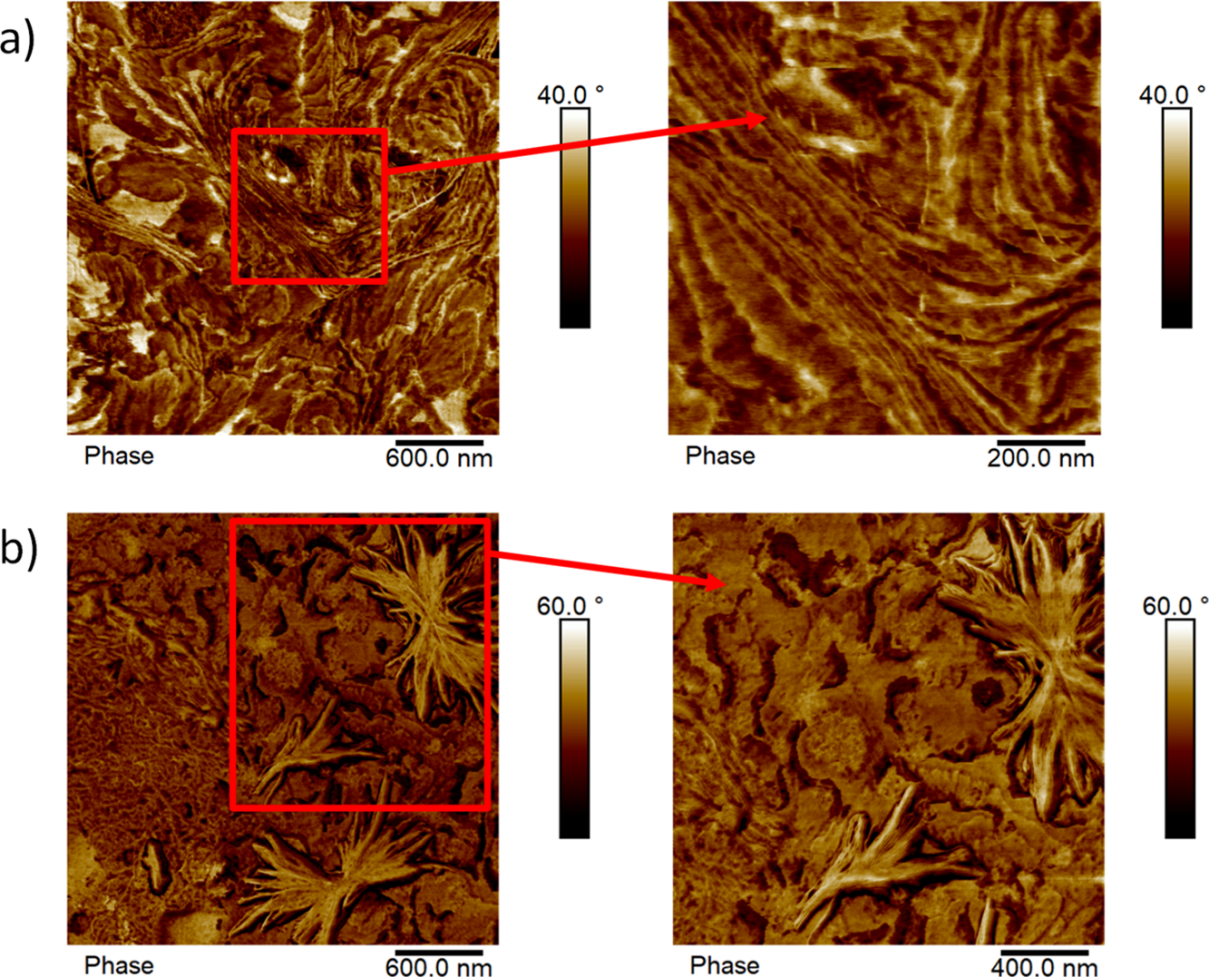
AFM micrographs of the tetrablock quarterpolymer PE_18_^7.1^*-b-*PEO_37_^15.1^*-b-*PCL_26_^10.4^*-b-*PLLA_19_^7.6^ (Q1) observed at 25 °C with close-ups of the indicated regions enclosed by a red box, applying two different thermal protocols: (a) cooling from the melt at 50 °C/min and (b) cooling from the melt at 20 °C/min.

The lamellar structure of the samples was analyzed by AFM employing two different thermal protocols to crystallize the blocks within the samples. The AFM phase micrographs correspond to the tetrablock quarterpolymer PE_18_^7.1^*-b-*PEO_37_^15.1^*-b-*PCL_26_^10.4^*-b-*PLLA_19_^7.6^ (Q1). The cooling rates employed in the preparation of the samples before obtaining these AFM micrographs at room temperature are the following: 50 °C/min for Figure 10a and 20 °C/min for Figure 10b. Parallel DSC experiments at both cooling rates demonstrated that all four blocks were able to crystallize, even the PLLA block at 50 °C/min (not shown here).

A close observation of the microstructure in these AFM micrographs makes it difficult to distinguish among the four crystalline blocks of the sample. It is very difficult to identify lamellae of different average thicknesses that correspond to each of the blocks. In addition, as some of the lamellae are edge-on, the calculation of an approximate value of average lamellar thickness is difficult. In the sample that was cooled at 20 °C/min, Figure 10b shows nascent spherulites with sizes below 1 μm composed of abundant radial lamellae that must correspond to lamellae of the four different crystalline components.

**Figure 11:**
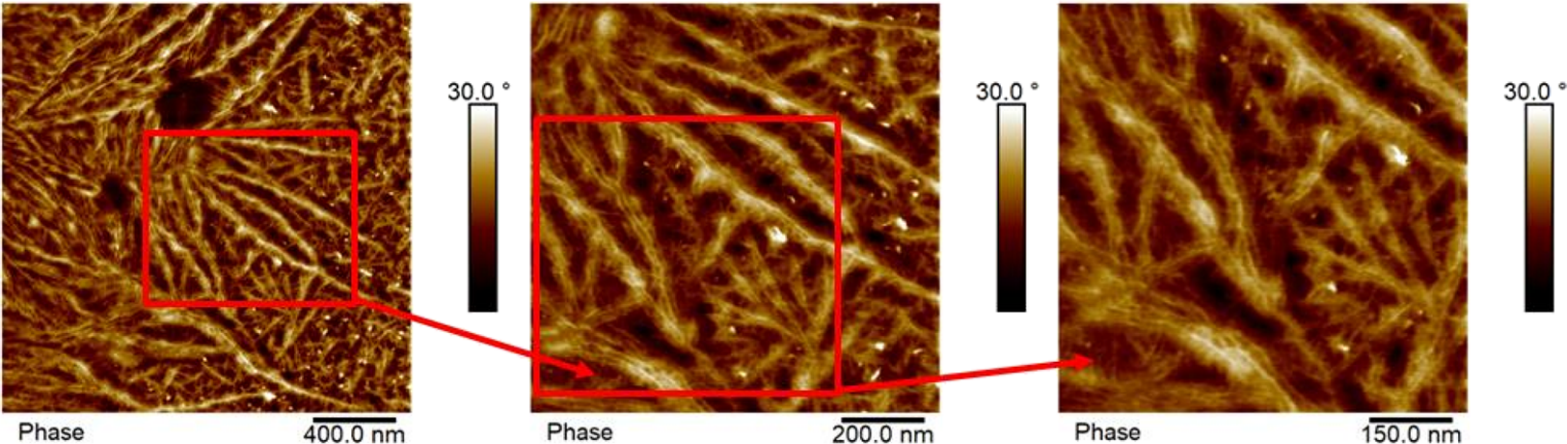
AFM micrographs of the tetrablock quarterpolymer PE_29_^9.5^*-b-*PEO_26_^8.8^*-b-*PCL_23_^7.6^*-b-*PLLA_22_^7.3^ (Q2) observed at 25 °C with close-ups of the indicated regions enclosed by a red box, using 20 °C/min as the cooling rate in the preparation of the sample.

**Figure 12:**
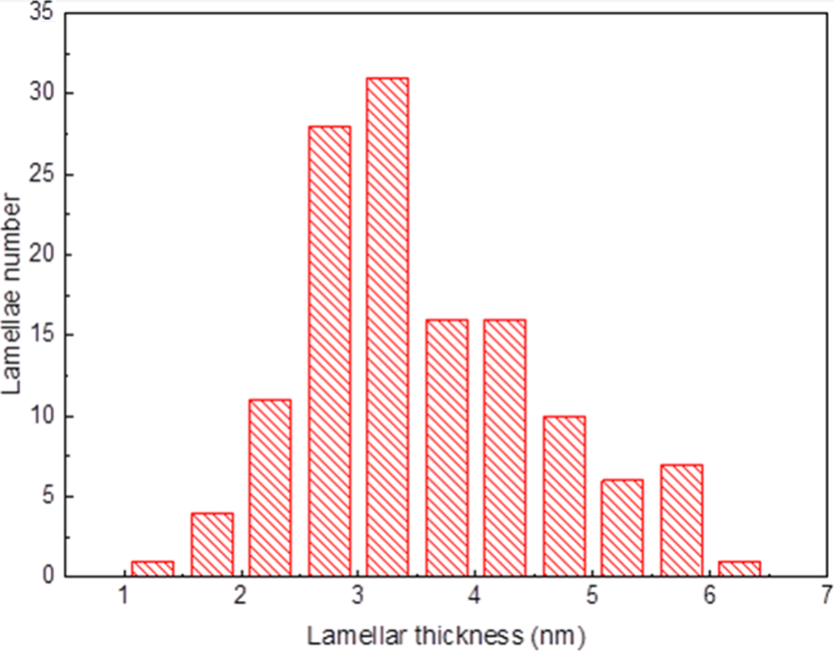
Lamellar thickness histogram obtained from the analysis of AFM micrographs shown in Figure 11.

Figure 11 shows AFM micrographs for the PE_29_^9.5^*-b-*PEO_26_^8.8^*-b-*PCL_23_^7.6^*-b-*PLLA_22_^7.3^ tetrablock quarterpolymer (Q2). The sample was prepared by using 20 °C/min as the cooling rate; no other rates were employed since no significant changes were obtained. The micrographs show the lamellar microstructure of the sample. The PLLA block in this tetrapolymer does not crystallize (Figures 2b, 3b, and 4b). Nevertheless, even with one less crystalline block than in the previous case (Figure 10), the distinction of the PE, PCL, and PEO crystalline lamellae remains complicated. An attempt to identify the three blocks was made by calculating the size of the lamellae detected in the micrographs, but as shown in Figure 12, a broad monomodal-like lamellar size distribution is obtained, making the differentiation of the three lamellar types impossible.

### 3.6 Mechanical Properties by Indentation

The above results show that the composition and molecular weight of the four blocks in the quarterpolymers strongly influence the course of lamellar development and the final lamellar nanostructure. In turn, such nanostructural differences are expected to influence the final properties of the material and, in particular, the mechanical properties. The storage modulus and hardness of the two tetrablock quarterpolymers were assessed by indentation, and results are collected in Table S9 of the Supporting Information (at an arbitrary indentation depth of 400 nm). Values of the ratio between the loss modulus and the storage modulus are also included. For the sake of comparison, Table S9 also includes data for the homopolymers, precursors, and one triblock copolymer with a high PLLA content.

Crystallinity values of all blocks were calculated from the DSC heating scans of samples prepared following the procedure described in the experimental section, and results are reported in Table S9. Note that as the crystallization and melting transitions of the PE and PLLA blocks on the one hand and the PEO and PCL blocks on the other hand overlap, an estimation of the individual crystallinities is quite a difficult task. For the PE and PLLA blocks, the crystallization and melting signals are bimodal, and we adopted an approximate determination of crystallinity by separating the enthalpy values of each block. In the case of PEO and PCL, an estimation of the crystallinity values is given by assuming an enthalpic contribution proportional to the content of each of the block.

**Figure 13:**
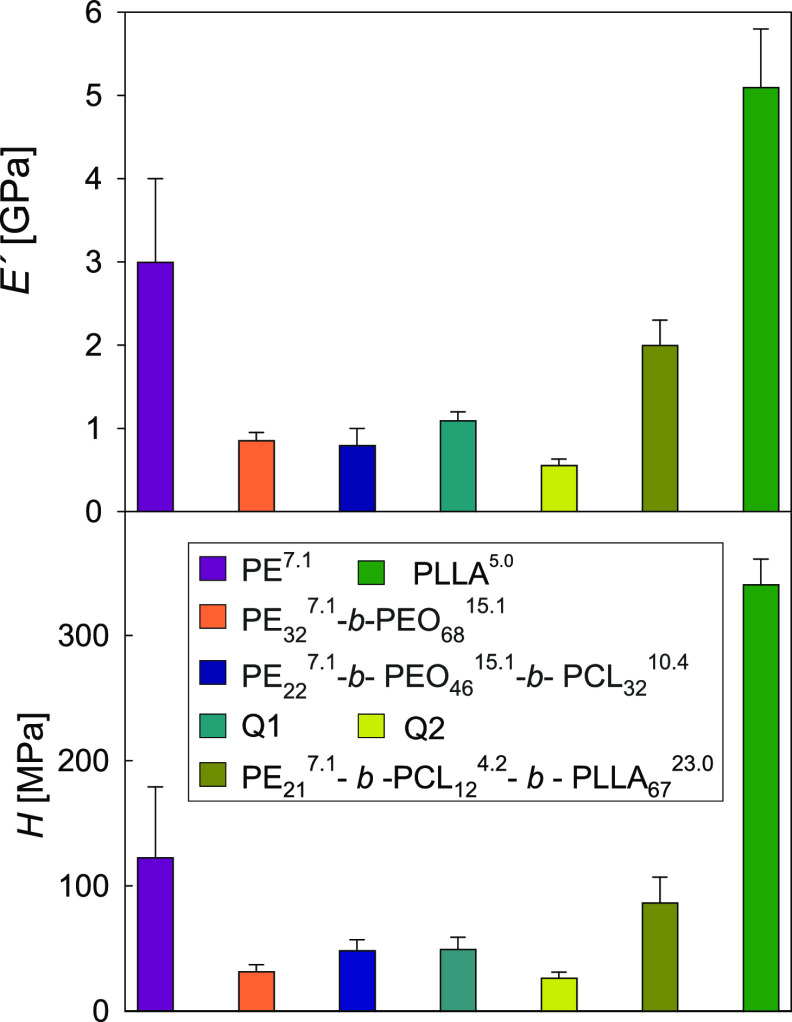
Storage modulus and hardness values (penetration depth = 400 nm) for the homopolymers, the two tetrablock quarterpolymers, the precursors including PE^7.1^, and one triblock copolymer with high PLLA content.

Figure 13 illustrates *E*′ and *H* data that serve as representative examples of the influence of composition and molecular weight on the mechanical behavior of the copolymers. The bars on the left- and the right-hand side of Figure 13 correspond to the PE^7.1^ and the PLLA^5.0^ homopolymers, respectively, and those in between relate to the copolymers. PE^7.1^ and PLLA^5.0^ display the highest *E*′ and *H* values of the four homopolymers (Table S9) and represent the ″hard″ blocks in the copolymers.

Table S9 shows that the higher-molecular-weight PE^9.5^ displays lower mechanical properties than PE^7.1^, and this can be attributed to the presence of thinner lamellar crystals, as suggested by the lower melting point of the 9500 g/mol material (i.e., melting point values of 124 and 108 °C for PE^7.1^ and PE^9.5^ blocks, respectively; see Table S9 in the Supporting Information). This can be attributed to the different molecular architectures of PE^7.1^ and PE^9.5^, as revealed by NMR and explained in the experimental part. In Q1, PE^7.1^ contains 0.32% propyl side groups and 3% methyl groups, while PE^9.5^ of Q2 has 0.45% propyl side groups and 2% methyl groups.

Figure 13 reveals that the incorporation of PEO blocks to PE^7.1^ produces a remarkable decrease of modulus and hardness values. This can be partially attributed to the lower mechanical properties of PEO that represents 68% of the molar fraction in the copolymer. However, in addition, PE crystallization is substantially hindered, and the low levels of crystallinity (8%) and the lamellar characteristics (*T*_m_ decreases by 12 °C with respect to the homopolymer) are also important factors that are expected to contribute to the *E*′ and *H* drop. Concurrent to this drop is the relevant change of *E″/E*′ ratio (see Table S9) that raises from 0.1 for PE^7.1^ to 0.18 for the diblock copolymer. This result suggests enhanced viscous behavior and can be related to the significant increase of the amorphous PE material.

The incorporation of PCL as a third block (PE_22_^7.1^*-b-*PEO_46_^15.1^*-b-*PCL_32_^10.4^) does not produce a substantial change of storage or loss modulus with respect to the diblock (Figure 13) because both PCL and PEO represent the compliant blocks in the terpolymer and, in addition, crystallinity levels of PE remain quite low (Table S9). However, a small *H*-increase is observed with the incorporation of the PCL block, and this could be related to the higher *H* values of PCL with respect to PEO.

Finally, Figure 13 shows that the addition of the fourth block to the terpolymer (Q1) does not produce a significant mechanical enhancement despite PLLA holding the highest *E*′ and *H* values of all blocks. This can be attributed to the low degree of crystallinity developed by PLLA (4%) while that of PE remains limited (7%, see Table S9). In contrast, the *E″/E*′ ratio significantly decreases (see Table S9), and this seems to be attributed to the contribution of the PLLA block that exhibits restricted viscous response. Concerning the Q2 copolymer, lower *E*′ and *H* values are found with respect to Q1 (Figure 13), and this could be explained as due to the inferior mechanical properties of the PE^9.5^ block in Q2 with respect to the PE^7.1^ one in Q1 (Table S9).

As a final point, the role of crystalline PLLA and PE can be clearly discerned with the triblock terpolymer PE_21_^7.1^*-b-*PCL_12_^4.2^*-b-*PLLA_67._^23^ In this case, both PE and PLLA exhibit significant crystallinity levels around 30–35%, which seem low compared to typical values for the homopolymers (Table S9) but appear to be enough to produce a clear *E*′ and *H* improvement (and a *E″/E*′ decrease, see Table S9) with respect to the terpolymer PE_22_^7.1^*-b-*PEO_46_^15.1^*-b-*PCL_32_^10.4^.

In summary, the mechanical properties of the tetrablock quarterpolymers and their precursors can be explained on the basis of the mechanical properties of the individual blocks, the block molar ratio, and the nanostructural characteristics arising after the crystallization process, such as the degree of crystallinity and the crystal lamellar thickness.

## 4 Conclusions

The analysis of the crystallization behavior in multiple block polymers becomes more complex as the number of potentially crystallizable blocks is increased. It is even more challenging if the temperature ranges at which crystallization and melting of more than one block overlaps. In this case, two tetracrystalline tetrablock quarterpolymers were studied, and we were able to clearly identify the crystallization and melting process of each individual block.

Both tetrablock quarterpolymers present small differences in composition and molecular weight of the blocks, as well as in the isotacticity percentage of PLLA. These differences are nevertheless significant, as the behavior of the two quarterpolymers examined is very different from one another. The PE_18_^7.1^*-b-*PEO_37_^15.1^*-b-*PCL_26_^10.4^*-b-*PLLA_19_^7.6^ (Q1) tetrablock quarterpolymer did not exhibit any phase segregation in the melt and was able to develop novel tetracrystalline spherulites upon cooling from the melt as all of its four blocks were able to crystallize. On the other hand, the PE_29_^9.5^*-b-*PEO_26_^8.8^*-b-*PCL_23_^7.6^*-b-*PLLA_22_^7.3^ (Q2) tetrablock quarterpolymer is characterized by presenting a weak lamellar phase segregation in the melt (as indicated by SAXS) and a breakout crystallization where the PLLA block cannot crystallize (low isotacticity). Therefore, for this material, the morphology consisted of tricrystalline microspherulites.

The use of synchrotron *in situ* WAXS, DSC, and PLOM (both observations and light intensity measurements) techniques was found to be essential to separate the overlapping crystallization processes of both PE/PLLA and PEO/PCL blocks and thus the sequence of crystallization of each of the four blocks within the quarter polymers.

The specific nanostructural features appearing as a result of the sequential crystallization of the blocks in the quarterpolymers are found to have a consequent impact on the mechanical properties. Storage modulus and hardness were assessed by nanoindentation, and it was found that both Q1 and Q2 exhibit relatively low *E*′ and *H* values (*E*′ ≤ 1 GPa, *H* ≤ 50 MPa) attributed to the small fraction of PE and PLLA crystals. Moreover, Q2 exhibits inferior mechanical properties than Q1, and this could be associated with the occurrence of thin PE crystal lamellae.

These complex tetrablock quarterpolymers containing apolar and biocompatible PE blocks and polar and biodegradable PEO, PCL, and PLLA blocks could find applications where their amphiphilic character could be useful, i.e., encapsulation and drug delivery, among others. From the academic point of view, it is remarkable that four different blocks can crystallize and self-assemble into highly ordered tetracrystalline negative spherulites that exhibit Maltese crosses and banding extinction patterns even though they are formed by at least four different lamellar types (e.g., in the case of Q1).

## Supporting Information Available

Segregation strength data; DSC cooling and heating scans at 20 °C/min of the precursors of the tetrablock quarterpolymers (PE^7.1^, PE_32_^7.1^*-b-*PEO_68_^15.1^, and PE_22_^7.1^*-b-*PEO_46_^15.1^*-b-*PCL_32_^10.4^; PE^9.5^, PE_52_^9.5^*-b-*PEO_48_^8.8^, and PE_37_^9.5^*-b-*PEO_34_^8.8^*-b-*PCL_29_^7.6^); DSC data tables of the tetrablock quarterpolymers PE_18_^7.1^*-b-*PEO_37_^15.1^*-b-*PCL_26_^10.4^*-b-*PLLA_19_^7.6^ (Q1) and PE_29_^9.5^*-b-*PEO_26_^8.8^*-b-*PCL_23_^7.6^*-b-*PLLA_22_^7.3^ (Q2); WAXS indexation data; WAXS patterns taken during subsequent heating at 20 °C/min for the tetrablock quarterpolymers Q1 and Q2; and PLOM heating micrographs at 20 °C/min of the tetrablock quarterpolymer PE_18_^7.1^*-b-*PEO_37_^15.1^*-b-*PCL_26_^10.4^*-b-*PLLA_19_^7.6^ (Q1) with PLOM intensity measurements (PDF)
