# Supplementary material for: Sequential Crystallization and Multicrystalline Morphology in PE-b-PEO-b-PCL-b-PLLA Tetrablock Quarterpolymers
Source: Macromolecules. 2021 Jul 23;54(15):7244–57. doi: 10.1021/acs.macromol.1c01186 (PMC9159653; doi:10.1021/acs.macromol.1c01186)
Supplement: Supplementary file 1 — ma1c01186_si_001.pdf [file ma1c01186_si_001.pdf]

Supporting Information

Sequential crystallization and multi-crystalline morphology in PE-*b*-PEO-*b*-PCL-*b*-PLLA tetrablock quarterpolymers

*Eider Matxinandiarena[1], Agurtzane Múgica[1], Agnieszka Tercjak[2], Viko Ladelta[3], George Zapsas[3], Nikos Hadjichristidis[3], Dario Cavallo[4], Araceli Flores[5], Alejandro J. Müller[1,6]**

[1]POLYMAT and Department of Polymers and Advanced Materials: Physics, Chemistry and Technology, University of the Basque Country UPV/EHU, Paseo Manuel Lardizábal 3, 20018, Donostia-San Sebastián, Spain.

[2]Group 'Materials + Technologies', Department of Chemical and Environmental Engineering, University of the Basque Country, UPV/EHU, Plaza Europa 1, 20018 Donostia-San Sebastián, Spain

[3]Polymer Synthesis Laboratory, KAUST Catalysis Center, Physical Sciences and Engineering Division, King Abdullah University of Science and Technology (KAUST), Thuwal, Saudi Arabia

[4]Department of Chemistry and Industrial Chemistry, University of Genova, via Dodecaneso 31, 16146 Genova, Italy

[5]Polymer Physics, Elastomers and Applications Energy, Institute of Polymer Science and Technology (ICTP-CSIC), Juan de la Cierva 3, 28006 Madrid, Spain

[6]Ikerbasque, Basque Foundation for Science, Plaza Euskadi 5, 48009 Bilbao, Spain.

Corresponding Author

*email: alejandrojesus.muller@ehu.es

Table S1. Values of χ and χN calculated for the tetrablock quarterpolymers at 180 °C

		$PE_{18}^{7.1}$ -*b* -$PEO_{37}^{15.1}$ -*b*- $PCL_{26}^{10.4}$ -*b*- $PLLA_{19}^{7.6}$	$PE_{29}^{9.5}$ -*b*- $PEO_{26}^{8.8}$ -*b*- $PCL_{23}^{7.6}$ -*b*- $PLLA_{22}^{7.3}$
	χ	χN	χN
PE-*b*-PEO	0.78	135	99
PE-*b*-PCL	0.59	37	36
PE-*b*-PLLA	0.74	54	63
PEO-*b*-PCL	0.37	31	19
PEO-*b*-PLLA	0.34	37	24
PCL-*b*-PLLA	0.36	18	14

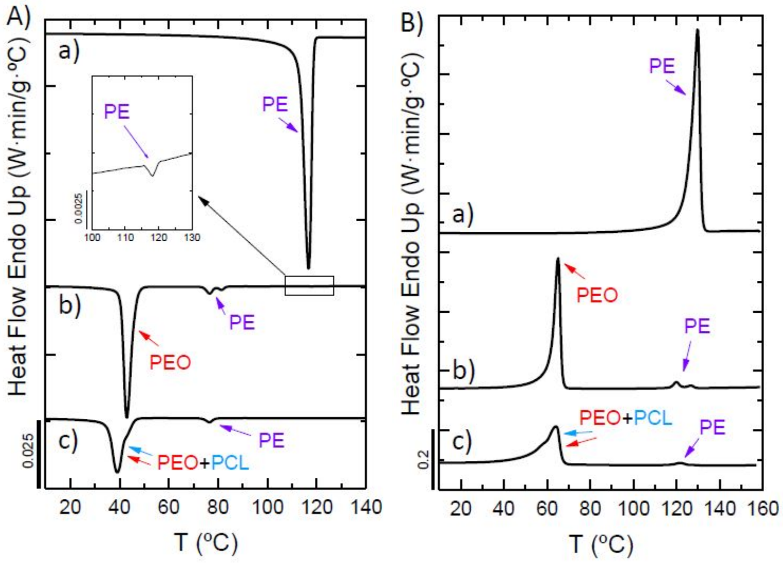

Figure S1. DSC scans at 20 ºC/min for a) $PE^{7.1}$, b) $PE_{32}^{7.1}$ -*b*- $PEO_{68}^{15.1}$ and c) $PE_{22}^{7.1}$ -*b*- $PEO_{46}^{15.1}$ -*b*- $PCL_{32}^{10.4}$ of A) cooling from the melt with a close-up to better identify PE crystallization in the diblock copolymer and B) subsequent heating with arrows indicating transitions for each block

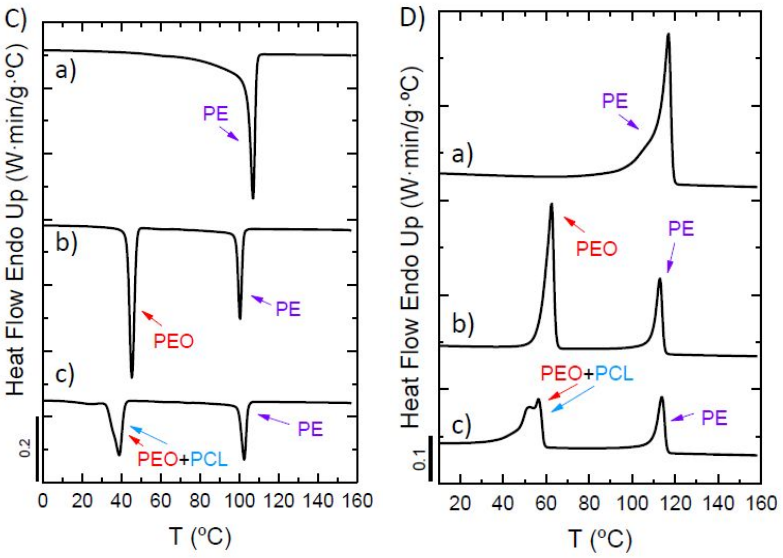

Figure S2. DSC scans for a) $PE^{9.5}$, b) $PE_{52}^{9.5}$ -*b*- $PEO_{48}^{8.8}$ and c) $PE_{37}^{9.5}$ -*b*- $PEO_{34}^{8.8}$ -*b*- $PCL_{29}^{7.6}$ of C) cooling from the melt and D) subsequent heating with arrows indicating transitions for each block

Table S2. Thermal DSC cooling properties of the homopolymers PE, diblock copolymers PE-*b*-PEO, and triblock terpolymers PE-*b*-PEO-*b*-PCL. Crystallization enthalpies are normalized according to block content in each of the samples

	$T_{c,\,PEO}$ (ºC)	$T_{c,\,PCL}$ (ºC)	$T_{c,\,PE}$ (ºC)	$\Delta H_{c,\,PEO}$ (J/g)	$\Delta H_{c,\,PCL}$ (J/g)	$\Delta H_{c,\,PE}$ (J/g)
**$PE^{7.1}$**	-	-	116.6	-	-	229.0
**$PE_{32}^{7.1}$ -*b*- $PEO_{68}^{15.1}$**	43.1	-	76.4<br>81.4<br>118.4	177.0<br>5	-	21.5
**$PE_{22}^{7.1}$ -*b*- $PEO_{46}^{15.1}$ -*b*- $PCL_{32}^{10.4}$**	39.1*		76.5	35.4	24.6	13.5
**$PE^{9.5}$**	-	-	107.2	-	-	156.2
**$PE_{52}^{9.5}$ -*b*- $PEO_{48}^{8.8}$**	45.2	-	100.5	177.0	-	80.9
**$PE_{37}^{9.5}$ -*b*- $PEO_{34}^{8.8}$ -*b*- $PCL_{29}^{7.6}$**	38.8*		102.5	21.0	17.9	113.4

*As crystallization temperatures of the PEO/PCL blocks are overlapped, a single T_c value is provided, and enthalpies are an estimation calculated according to block content

Table S3. Thermal DSC heating properties of the homopolymers PE, diblock copolymers PE-*b*-PEO, and triblock terpolymers PE-*b*-PEO-*b*-PCL. Melting enthalpies are normalized according to block content in each of the samples

	$T_{m, PEO}$ (ºC)	$T_{m, PCL}$ (ºC)	$T_{m, PE}$ (ºC)	$\Delta H_{m, PEO}$ (J/g)	$\Delta H_{m, PCL}$ (J/g)	$\Delta H_{m, PE}$ (J/g)
**$PE^{7.1}$**	-	-	129.7	-	-	224.8
**$PE_{32}^{7.1}$ -*b*- $PEO_{68}^{15.1}$**	65.1	-	120.0<br>126.7	181.5	-	20.8
**$PE_{22}^{7.1}$ -*b*- $PEO_{46}^{15.1}$ -*b*- $PCL_{32}^{10.4}$**	64.2*		122.0	38.7	26.9	13.1
**$PE^{9.5}$**	-	-	117.0	-	-	160.2
**$PE_{52}^{9.5}$ -*b*- $PEO_{48}^{8.8}$**	62.6	-	113.0	184.6	-	78.8
**$PE_{37}^{9.5}$ -*b*- $PEO_{34}^{8.8}$ -*b*- $PCL_{29}^{7.6}$**	51.4	56.3	113.7	22.8	19.4	127.6

*As crystallization temperatures of the PEO/PCL blocks are overlapped, a single value is provided, and the corresponding enthalpies are an estimation calculated according to block content

Table S4. Crystallinity values (%) of the samples calculated from DSC heating scans taking into account the mass fractions of each of the blocks and using $X_c=(\Delta H_m/\Delta H_{m,100\%})\cdot 100$ and enthalpy of fusion of 100% crystalline polymers ($\Delta H_{m,100\%}$) is taken from literature: 293 J/g for PE[1], 139 J/g for PCL[2] and 214 J/g for PEO[3]

	$X_{c,\,PEO}$ (%)	$X_{c,\,PCL}$ (%)	$X_{c,\,PE}$ (%)
**$PE^{7.1}$**	-	-	76.7
**$PE_{32}^{7.1}$ -*b*- $PEO_{68}^{15.1}$**	84.8	-	7.1
**$PE_{22}^{7.1}$ -*b*- $PEO_{46}^{15.1}$ -*b*- $PCL_{32}^{10.4}$**	39.3*	60.4*	4.5
**$PE^{9.5}$**	-	-	54.7
**$PE_{52}^{9.5}$ -*b*- $PEO_{48}^{8.8}$**	86.2	-	26.9
**$PE_{37}^{9.5}$ -*b*- $PEO_{34}^{8.8}$ -*b*- $PCL_{29}^{7.6}$**	31.3*	48.1*	43.5

*PEO and PCL crystallinity values in the triblock terpolymers are estimation according to block content since the melting peaks of the blocks are overlapped and melting enthalpies cannot be distinguished.

Table S5. Thermal DSC cooling properties of the tetrablock quarterpolymers Q1) $PE_{18}^{7.1}$ -*b* -$PEO_{37}^{15.1}$ -*b*- $PCL_{26}^{10.4}$ -*b*- $PLLA_{19}^{7.6}$; Q2) $PE_{29}^{9.5}$ -*b*- $PEO_{26}^{8.8}$ -*b*- $PCL_{23}^{7.6}$ -*b*- $PLLA_{22}^{7.3}$ (the PLLA block does not crystallize)

	T_c, PEO (ºC)	T_c, PCL (ºC)	T_c, PE (ºC)	T_c, PLLA (ºC)	ΔH_c, PEO (J/g)	ΔH_c, PCL (J/g)	ΔH_c, PE (J/g)	ΔH_c, PLLA (J/g)
**Q1**	36.4	43.8	75.1	81.2	33.5	23.6	3.6	1.7
**Q2**	25.5		100.5	-	25.7	22.7	46.8	-

Table S6. Thermal DSC heating properties of the tetrablock quarterpolymers Q1) $PE_{18}^{7.1}$ -*b* -$PEO_{37}^{15.1}$ -*b*- $PCL_{26}^{10.4}$ -*b*- $PLLA_{19}^{7.6}$; Q2) $PE_{29}^{9.5}$ -*b*- $PEO_{26}^{8.8}$ -*b*- $PCL_{23}^{7.6}$ -*b*- $PLLA_{22}^{7.3}$ (the PLLA block does not crystallize)

	$T_{m,}$ PEO (ºC)	$T_{m,}$ PCL (ºC)	$T_{m,}$ PE (ºC)	$T_{m,}$ PLLA (ºC)	$\Delta H_{m,}$ PEO (J/g)	$\Delta H_{m,}$ PCL (J/g)	$\Delta H_{m,}$ PE (J/g)	$\Delta H_{m,}$ PLLA (J/g)
**Q1**	56.0	60.8	122.8	132.5	37.5	26.4	2.3	1.9
**Q2**	50.7		114.7	-	28.2	24.9	42.4	-

Table S7. Crystallinity values (%) of the samples calculated from DSC cooling and heating data taking into account the mass fractions of each of the blocks and using $X_c=(\Delta H_m/\ \Delta H_{m,100\%})\cdot 100$ and enthalpy of fusion of 100% crystalline polymers ($\Delta H_{m,100\%}$) is taken from literature: 293 J/g for PE[1], 139 J/g for PCL[2], 214 J/g for PEO[3] and 93 J/g for PLLA[4]

		$X_{c, PEO}$ (%)	$X_{c, PCL}$ (%)	$X_{c, PE}$ (%)	$X_{c, PLLA}$ (%)
**Q1**	Cooling	65	42	6	9
	Heating	41	70	4	11
**Q2**	Cooling	46	71	55	-
	Heating	51	78	50	-

*Tetrablock quarterpolymers: Q1) $PE_{18}^{7.1}$ -*b* -$PEO_{37}^{15.1}$ -*b*- $PCL_{26}^{10.4}$ -*b*- $PLLA_{19}^{7.6}$; Q2) $PE_{29}^{9.5}$ -*b*- $PEO_{26}^{8.8}$ -*b*- $PCL_{23}^{7.6}$ -*b*- $PLLA_{22}^{7.3}$

*As cooling and heating DSC transitions of the blocks PE/PLLA and PEO/PCL overlap, an estimation of the crystallinity values according to block content is reported. The PLLA block in Q2 does not crystallize.

Table S8. WAXS indexation of the PE, PLLA, PCL and PEO blocks of the samples[5-13]

Blocks	(hkl) planes	q values (nm^{-1})
**PLLA**	(010)	10.3
**PLLA**	(110)/(200)	12.0
**PLLA**	(113)/(203)	13.5
**PEO**	(120)	13.8
**PE**	(110)	15.4
**PCL**	(110)	15.5
**PLLA**	(210)	15.7
**PEO**	(032)/(112)/(132)/(212)	16.4
**PCL**	(200)	16.7
**PE**	(200)	16.9

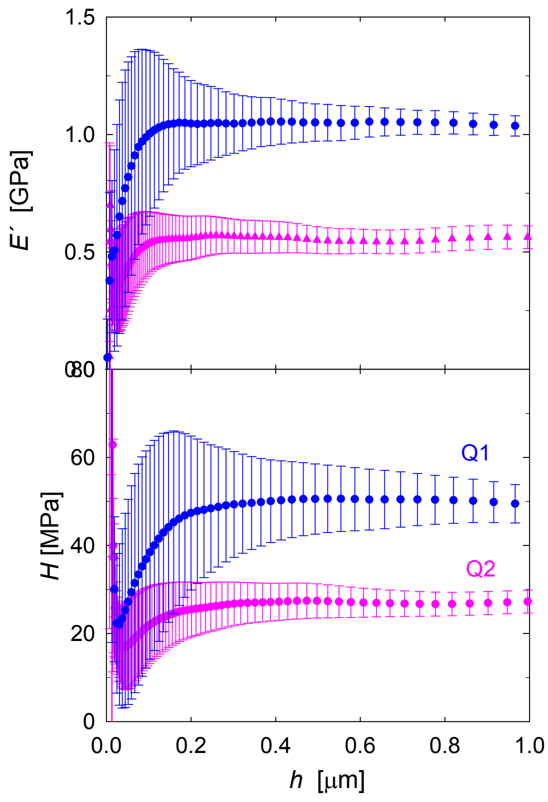

Figure S3. Variation of E' and H with indentation depth, h, for Q1 and Q2. E' and H values represent the average of 70 and 50 indentation tests for Q1 and Q2, respectively. Data at small penetration depths below h = 300 nm can be disregarded because they are affected by surface roughness and material independent effects such as tip area miscalibrations[14]

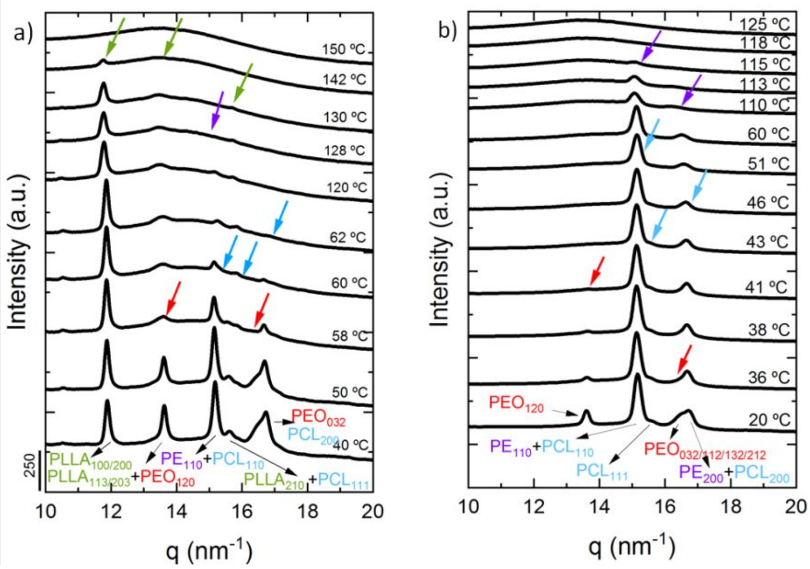

Figure S4. WAXS patterns taken during subsequent heating at 20 °C/min for a) $PE_{18}^{7.1}$ -*b* -$PEO_{37}^{15.1}$ -*b*- $PCL_{26}^{10.4}$ -*b*- $PLLA_{19}^{7.6}$ and b) $PE_{29}^{9.5}$ -*b*- $PEO_{26}^{8.8}$ -*b*- $PCL_{23}^{7.6}$ -*b*- $PLLA_{22}^{7.3}$, at different temperatures with arrows indicating transitions for each block (violet for PE, green for PLLA, blue for PCL and red for PEO) and the corresponding (hkl) planes of the blocks

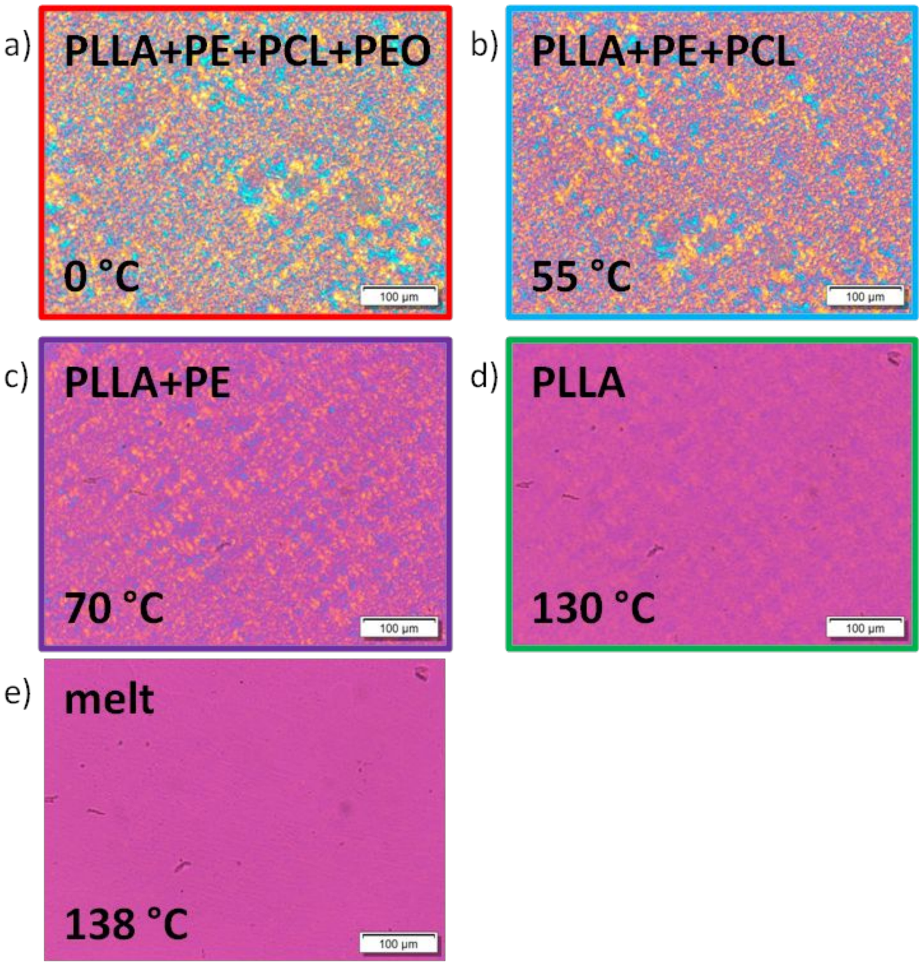

Figure S5. PLOM subsequent heating micrographs from 0 ºC to the melt at 20 °C/min for the tetrablock quarterpolymer $PE_{18}^{7.1}$ -*b* -$PEO_{37}^{15.1}$ -*b*- $PCL_{26}^{10.4}$ -*b*- $PLLA_{19}^{7.6}$ (Q1) with colored boxes indicating the crystallization of each of the blocks (violet for PE, green for PLLA, blue for PCL and red for PEO): a) PLLA, PE, PCL and PEO at 0 ºC, b) PLLA, PE and PCL at 55 ºC, c) PLLA and PE at 70 ºC, d) PLLA at 130 ºC, and e) the molten state at 138 ºC

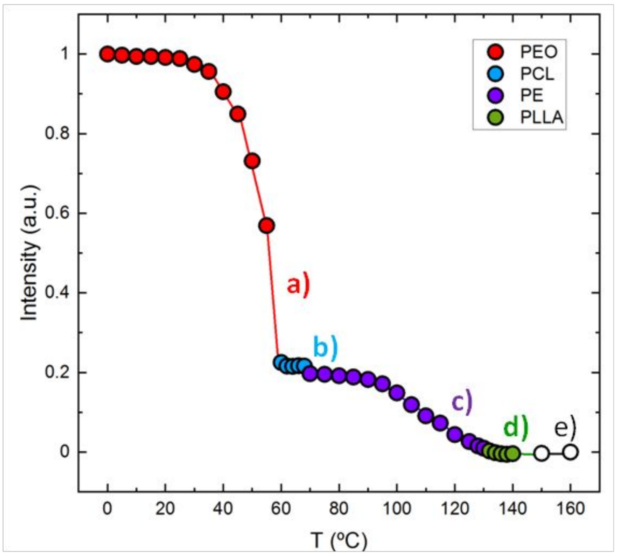

Figure S6. PLOM intensity measurements from micrographs of Figure S5 as a function of temperature indicating melting of the a) PEO block, b) PCL block, c) PE block, d) PLLA block and e) melting for the tetrablock quarterpolymer $PE_{18}^{7.1}$ –*b* –$PEO_{37}^{15.1}$ –*b*-$PCL_{26}^{10.4}$ –*b*- $PLLA_{19}^{7.6}$ (Q1) with coloured data points and lines (red for PEO, blue for PCL, violet for PE and green for PLLA) to follow crystallization of each block. Empty data points represent the molten state of the sample

Table S9. Indentation storage modulus and hardness hardness and ratio between loss and storage modulus for the two tetrablock quarterpolymers and their precursors. Values for one triblock terpolymer including a PLLA block are also included for the sake of comparison. For PEO, the size of the indentations was significantly lower than the spherulites radius and hence, *E´* and *H* data do not represent the mean values but just the range of experimental mechanical properties determined at different locations. Melting points and crystallinity values are calculated from DSC heating scans.

Sample	*E´* (GPa)	*H* (MPa)	*E´´/E´*	X_c (%)	T_m (ºC)
$PE^{7.1}$	3 ± 1	123± 56	0.10	78	124^{PE}
$PE_{32}^{7.1}$-*b*-$PEO_{68}^{15.1}$	0.86 ± 0.09	32 ± 5	0.18	8^{PE} 85^{PEO}	$112,120^{PE}$ 49^{PEO}
$PE_{22}^{7.1}$-*b*-$PEO_{46}^{15.1}$-*b*-$PCL_{32}^{10.4}$	0.8 ± 0.2	49 ± 8	0.17	4^{PE} 49^{PEO}	$114,120^{PE}$ $46^{PEO+PCL}$

				76^{PCL}	
$PE_{21}^{7.1}$-*b*-$PCL_{12}^{4.2}$-*b*-$PLLA_{67}^{23}$	2.0 ± 0.3	87 ± 20	0.12	31^{PE}<br>67^{PCL}<br>36^{PLLA}	119^{PE}<br>40^{PCL}<br>158^{PLLA}
$PE_{18}^{7.1}$-*b*-$PEO_{37}^{15.1}$-*b*-$PCL_{26}^{10.4}$-*b*-$PLLA_{19}^{7.6}$ (Q1)	1.1 ± 0.1	50 ± 9	0.14	7^{PE}<br>44^{PEO}<br>69^{PCL}<br>4^{PLLA}	117^{PE}<br>136^{PLLA}<br>$44^{PEO+PCL}$
$PE^{9.5}$	1.8 ± 0.2	58 ±7	0.12	62	108^{PE}
$PE_{52}^{9.5}$-*b*-$PEO_{48}^{8.8}$	0.96 ± 0.07	57± 6	0.13	35^{PE}<br>82^{PEO}	106^{PE}<br>48^{PEO}
$PE_{37}^{9.5}$-*b*-$PEO_{34}^{8.8}$-*b*-$PCL_{29}^{7.6}$	0.64 ± 0.05	28 ± 4	0.16	46^{PE}<br>48^{PEO}<br>74^{PCL}	107^{PE}<br>$40^{PEO+PCL}$
$PE_{29}^{9.5}$-*b*-$PEO_{26}^{8.8}$-*b*-$PCL_{23}^{7.6}$-*b*-$PLLA_{22}^{7.3}$ (Q2)	0.56 ± 0.07	27 ± 4	0.17	51^{PE}<br>57^{PEO}<br>88^{PCL}<br>0^{PLLA}	109^{PE}<br>$39^{PEO+PCL}$
PEO^{20}	0.25 – 1.5	46 – 80		79	55^{PEO}
$PCL^{8.4}$	1.2 ± 0.2	91 ± 11	0.09	55	44^{PCL}
$PLLA^{5.0}$	5.1 ± 0.7	341 ± 20	0.067	71	157^{PLLA}

REFERENCES

1. Ren, M.; Tang, Y.; Gao, D.; Ren, Y.; Yao, X.; Shi, H.; Zhang, T.; Wu, C., Recrystallization of biaxially oriented polyethylene film from partially melted state within crystallite networks. *Polymer* **2020,** *191*, 122291.

2. Izquierdo, R. G.-G., N.; Rodríguez, M. T.; Cáceres, E.; García, S. J.; Gómez Ribelles, J. M.; Monleón, M.; Monllau, J. C.; Suay, J., Biodegradable PCL scaffolds with an interconnected spherical pore network for tissue engineering. *Journal of Biomedical Materials Research* **2007**, 25-35.

3. Ebers, L. S.; Auvergne, R.; Boutevin, B.; Laborie, M. P., Impact of PEO structure and formulation on the properties of a Lignin/PEO blend. *Industrial Crops and Products* **2020,** *143*, 111883.

4. Shamsah, A. H.; Cartmell, S. H.; Richardson, S. M.; Bosworth, L. A., Material characterization of PCL: PLLA electrospun fibers following six months degradation in vitro. *Polymers* **2020,** *12*, 700.

5. Huang, C. I.; Tsai, S. H.; Chen, C. M., Isothermal crystallization behaviour of poly(L-lactide) in poly(L-lactide)-*block*-poly(ethylene glycol) diblock copolymers. *Journal of Polymer Science: Part B: Polymer Physics* **2006,** *44*, 2438-2448.

6. Jiang, S.; He, C.; An, L.; Chen, X.; Jiang, B., Crystallization and ring-banded spherulite morphology of poly(ethylene oxide)-*block*-poly(E-caprolactone) diblock copolymer. *Macromolecular Chemistry and Physics* **2004,** *205*, 2229-2234.

7. Palacios, J. K.; Mugica, A.; Zubitur, M.; Iturrospe, A.; Arbe, A.; Liu, G.; Wang, D.; Zhao, J.; Hadjichristidis, N.; Müller, A. J., Sequential crystallization and morphology of triple crystalline biodegradable PEO-*b*-PCL-*b*-PLLA triblock terpolymers. *Royal Society of Chemistry Adv.* **2016,** *6*, 4739.

8. Maglio, G.; Migliozzi, A.; Palumbo, R., Thermal properties of di- and triblock copolymers of poly (L-lactide) with poly(oxyethylene) or poly (E-caprolactone). *Polymer* **2003,** *44*, 369-375.

9. Hamley, I. W.; Castelletto, V.; Castillo, R. W.; Müller, A. J.; Martin, C. M.; Pollet, E.; Dubois, P., Crystallization in poly(L-lactide)-*b*-poly(e-caprolactone) double crystalline diblock copolymers: a study using X-ray scattering, differential scanning calorimetry and polarized optical microscopy. *Macromolecules* **2005,** *38*, 463-472.

10. Hamley, I. W.; Parras, P.; Castelletto, V.; Castillo, R. V.; Müller, A. J.; Pollet, E.; Dubois, P.; Martin, C. M., Melt structure and its transformation by sequential crystallization of the two blocks within poly(L-lactide)-*block*- poly(e-caprolactone) double crystalline diblock copolymers. *Macromolecular Chemistry and Physics* **2006,** *207*, 941-953.

11. Huang, S.; Jiang, S.; An, L.; Chen, X., Crystallization and morphology of poly(ethylene oxide-*b*-lactide) cristalline-crystalline diblock copolymers. *Journal of Polymer Science, Part B: Polymer Physics* **2008,** *46*, 1400-1411.

12. Wang, J. L.; Dong, C. M., Synthesis, sequential crystallization and morphological evolution of well-defined star-shaped poly(e-caprolactone)-*b*-poly(L-lactide) block copolymer. *Macromolecular Chemistry and Physics* **2006,** *207*, 554-562.

13. Zhang, J.; Tashiro, K.; Tsuji, H.; Domb, J., Disorder-to-Order Phase Transition and Multiple Melting Behavior of Poly(L-lactide) Investigated by Simultaneous Measurements of WAXD and DSC. *Macromolecules* **2008,** *41*, 1352-1357.

14. Díez-Pascual, A. M.; Gómez-Fatou, M. A.; Ania, F.; Flores, A., Nanoindentation in polymer nanocomposites. *Progress in Materials Science* **2015,** *67*, 1-94.
